# High browsing skeletal adaptations in *Spinophorosaurus* reveal an evolutionary innovation in sauropod dinosaurs

**DOI:** 10.1038/s41598-020-63439-0

**Published:** 2020-04-20

**Authors:** D. Vidal, P. Mocho, A. Aberasturi, J. L. Sanz, F. Ortega

**Affiliations:** 1 Grupo de Biología Evolutiva, Facultad de Ciencias, UNED, Paseo Senda Del Rey, 9, 28040 Madrid, Spain; 2 Instituto Dom Luiz, Universidade de Lisboa, Bloco C6, 38Piso, sala 6.3.57, Campo Grande, 1749-016 Lisbon, Portugal; 3 The Dinosaur Institute, Natural History Museum of Los Angeles County, 900 Exposition Blvd., 90007 CA Los Angeles, USA; 4 Museo Paleontológico de Elche, Carrer Sant Joan, 3, 03203 Elche, Spain; 5 Unidad de Paleontología, Facultad de Ciencias, Universidad Autónoma de Madrid, Calle Darwin, 2, 28049 Madrid, Spain; 6 Real Academia Española de Ciencias Exactas, Físicas y Naturales, Calle Valverde, 24, 28004 Madrid, Spain

**Keywords:** Palaeontology, Biomechanics

## Abstract

Sauropods were among the most diverse lineages of dinosaurs, with an ample geographic distribution throughout the Mesozoic. This evolutionary success is largely attributed to neck elongation and its impact on feeding efficiency. However, how neck elongation influenced exactly on feeding strategies is subject of debate. The process of mounting a nearly complete virtual skeleton of *Spinophorosaurus nigerensis*, from the Middle (?) Jurassic of Niger, has revealed several previously unknown osteological adaptations in this taxon. Wedged sacral and posterior dorsal vertebrae cause the presacral column to deflect antero-dorsally. This, together with elongated scapulae and humeri make the anterior region of the skeleton vertically lengthened. Also, elongated prezygapophyseal facets on the cervical vertebrae and a specialized first dorsal vertebra greatly increase the vertical range of motion of the neck. These characters support this early eusauropod as a more capable high browser than more basally branching sauropods. While limb proportions and zygapophyseal facets vary among Eusauropoda, the sacrum retained more than 10° of wedging in all Eusauropoda. This implied a functional constraint for sauropod species which evolved lower browsing feeding strategies: the antero-dorsal sloping caused by the sacrum had to be counteracted with further skeletal modifications, e.g. a ventrally curved mid to anterior presacral spine to hinder the dorsal slope of the whole presacral series caused by the wedged sacrum. This suggests that at least the last common ancestor of Eusauropoda developed high browsing capabilities, partially due to the modified wedged sacrum, likely a potential synapomorphy of the clade and key in the evolutionary history of the group.

## Introduction

Sauropods were the earliest large phytophagous dinosaurs, with an unparalleled disparity in body size, since their Late Triassic origin until their demise at the end of the Cretaceous^1^. Their quadrupedal, long-necked, long-tailed body plan remained fixed during their evolution, although both relatively shorter necks^2,3^ and extremely long necks^4–7^ appeared on several different clades of sauropods. This characteristic body plan had a direct impact on the feeding efficiency of these animals^1,8^, with some changes on this body plan being likely feeding adaptations, key in their evolutionary history^8^. However, whether these adaptations allowed or not high browsing capabilities has been the subject of a lively debate^9–15^. Previous studies on sauropod feeding capabilities based on their postcrania have focused mostly on neck posture and range of motion^6,9,11,12,15–17^. Evidence from neutral articulation of the bones and computerized analyses suggested straighter, less flexible necks^9,14,16^, with some authors suggesting most sauropods could barely raise the neck above shoulder height^9,16^. However, evidence stemming from comparisons with extant relatives and analyses on inter-vertebral stress suggested elevated, more flexible and curved necks^10–13,18^. More recent studies have revealed that the relationship between sauropod neck posture and feeding habits is more complex than previously thought^14,19^: forelimb/hindlimb proportions^15^ or scapula orientation and position probably had a strong role in browsing capabilities as well^14,20^. Unfortunately, the fragmentary nature of the known sauropod fossil record has been a large caveat in understanding how the axial skeleton, girdles and limbs vary within sauropod dinosaurs, making the study of their functional morphology complex.

In 2007, the holotypic specimen of *Spinophorosaurus nigerensis* was unearthed from the Middle (?) Jurassic of Niger^21,22^, being one of the most complete single specimens retrieved among basally branching eusauropods. The exceptional preservation and completeness of this specimen enabled to generate a virtual skeletal mount with less uncertainty than previous virtual sauropod mounts. A high-resolution virtual skeleton was used to test (i) what the body plan and feeding capabilities of *Spinophorosaurus* were, (ii) previous claims on sauropod neck functional morphology and (iii) whether previous reconstructions were or not accurate.

## Results

### *Spinophorosaurus* body plan

**Figure 1:**
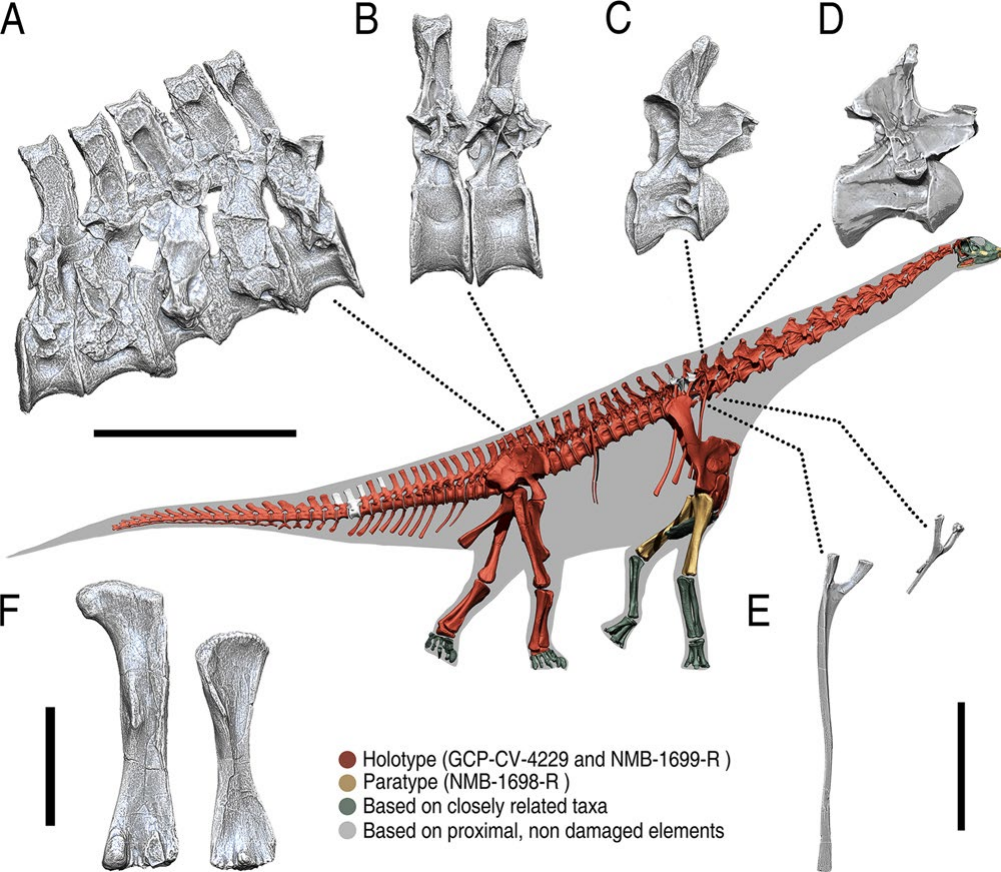
High browsing adaptations in *Spinophorosaurus nigerensis*. Skeletal reconstruction in osteologically neural pose with bones color-coded according to their provenance, with the holotype in red, paratype in yellow, and bones inferred from close relatives in green. White indicates reconstructed bone. (**A**) Sacrum, showing the 20° angle wedging. (**B**) 12^th^ and 13^th^ dorsal vertebrae, showing a slight acute wedging. (**C**) Partially cervicalized 1^st^ dorsal vertebra. (**D**) 12^th^ Cervical Vertebra. (**E**) First dorsal rib and last cervical rib. (**F**) Humerus and femur, to scale. Scales A-F = 500 mm.

Mounting the digital skeleton has revealed *Spinophorosaurus* had tall shoulders and an elevated neck, well above shoulder level, in osteologically induced curvature (OIC; Fig. 1, see Terminology in SI *appendix*, Section 1). The OIC of *Spinophorosaurus* is the result of articulating a skeleton in osteologically neutral pose (ONP, maximum zygapophyseal overlap, see Material and Methods below) with only two vertebrae visible at once. This makes the mount more a product of bone geometry alone, with preconceived notions minimized^14,23^. The resulting body plan, analogous to that of the more extremely verticalized brachiosaurid sauropods, is due to two main skeletal characters: (i) an elongated forelimb, the humerus being longer relative to the scapula and femur than those of most other sauropods, and the scapula being slightly longer than the femur (Table 1); and (ii) an acute wedged sacrum and posteriormost dorsal vertebrae, which deflect the presacral vertebrae dorsally in neutral articulation (Fig. 1).

**Table 1:** Length measurements and ratios of different sauropod taxa, with specimen number indicated.

Taxa	Sc Length	H Length	F Length	F/H Ratio	Sacrum Angle
*Spinophorosaurus nigerensis* GCP-CV-4229	1243	1014*	1215	1.20	20.1
*Shunosaurus lii* ZDM T5402	902	670	1200	1.79	14.7
*Mamenchisaurus youngi* ZDM0083	1190	825	1160	1.40	15.8
*Omeisaurus tianfuensis* ZDM T5704	1330	1040	1280	1.23	18.5
*Jobaria tiguidensis* MNN TIG4	?	1040*	1800	1.73	15.0
*Dicraeosaurus hansemanni* MB.R.4886	?	620 *	1220	1.96*	11.0
*Diplodocus carnegii*. CM94	1240	?	1542	?	16.0
*Apatosaurus louisae* CM3018	1640	1150	1785	1.55	15.5
*Brontosaurus excelsus* YPM 1980	?	1114	1910	1,72	15.0
*Tehuelchesaurus benitezii* MPEF-PV-1125	1801	1183	1550	1.31	20?
*Camarasaurus grandis* YPM 1901	1.155	856	1172	1.37	17.5
*Brachiosaurus altithorax* FMNH P25107	?	2042	2025	0.99	28.8
*Giraffatitan brancai* MB.R 2181 (formerly SII)	1930	2130	1990*	0.93	25.0
*Dreadnoughtus schrani* MPM PV 1156	1772	1600	1910	1,19	21.1
*Opisthocoelicaudia skarzynski* ZPAL MgD1/48	1180	1000	1395	1,39	14.1
*Kotasaurus yamanpalliensis*	?	*770	1130	1,46*	3.7
*Melanorosaurus readi* NM QR1551	489	450	638	1,41	1.0
*Lufengosaurus huenei* IVPP 15	?	335	560	1.67	5.4
*Jingshanosaurus xinwaensis* LFGT-ZLJ0113	?	470	850	1.80	1.5
*Yunnanosaurus huangi* IVPP V20	305	231	435	1,88	2.1

**Figure 2:**
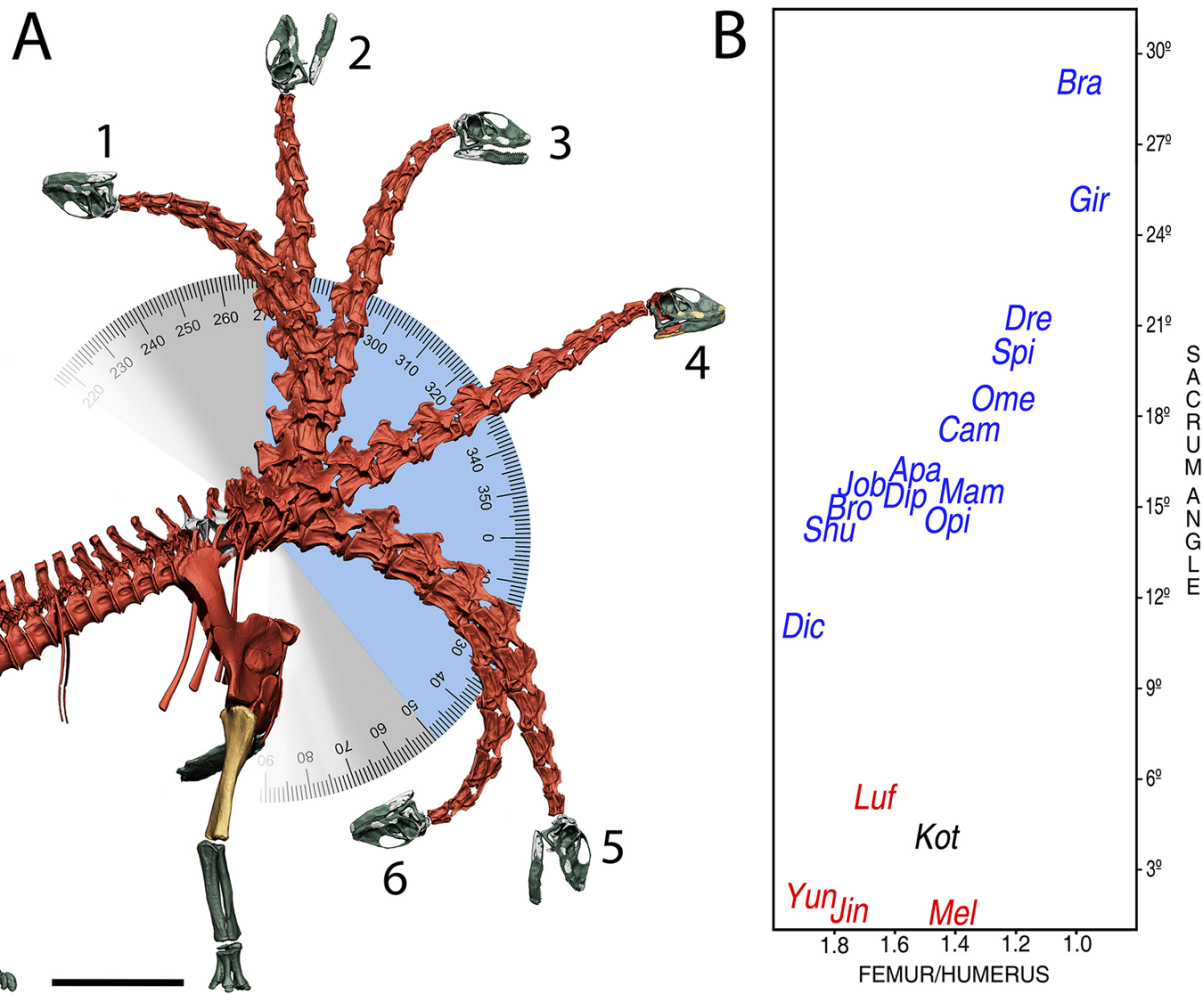
The verticalization of sauropod feeding envelopes. (**A**) Increased neck range of motion in *Spinophorosaurus* in the dorso-ventral plane, with the first dorsal vertebra as the vertex and 0° marking the ground. Poses shown: (1) maximum dorsiflexion; (2) highest vertical reach of the head (7.16 m from the ground), with the neck 90° deflected; (3) alert pose sensu Taylor Wedel and Naish^13^; (4) osteological neutral pose sensu Stevens^14^*;* (5) lowest vertical reach of the head (0.72 m from the ground at 0°), with the head as close to the ground without flexing the appendicular elements; (6) maximum ventriflexion. Blue indicates the arc described between maximum and minimum head heights. Grey indicates the arc described between maximum dorsiflexion and ventriflexion. (**B**) Bivariant plot comparing femur/humerus proportion with sacrum angle. The proportion of humerus and femur are compared as a ratio of femur maximum length/humerus maximum length. Sacrum angle measures the angle the presacral vertebral series are deflected from the caudal series by sacrum geometry in osteologically neutral pose. Measurements and taxa on Table 1. Scale = 1000 mm.

Asterisk (*) indicates estimated measurements scaled from other specimens of the same species (see Supplementary Material). The sacrum angle and F/H ratio are plotted in the XY graphic on Fig. 2B. All lengths in mm. Angles in degrees. Sc = scapula, H = humerus, F = femur.

Given the position of the pelvis in Sauropoda, with an antero-posteriorly projected ilium and a mesopubic and opisthoischiatic condition^24^ (which allows the femur to be upright and graviportal), the coalesced sacrum is situated so that the posterior face of the last sacral centrum is sub-vertical, making the tail sub-horizontal and the presacral series to slope antero-dorsally in ONP. This way, when the last dorsal vertebra articulates with the sacrum, it deflects 20° from the centrum of the first caudal vertebra in lateral view (Fig. S4). In addition, the slightly wedged centra of the two last dorsal vertebrae (Fig. 1B) deflect the dorsal series further, making the first dorsal vertebra deflect 5° from the last dorsal centrum (overall 25° from the first caudal). The cervical series is almost straight, but with a slight sigmoidal dorsal deflection, with the axis deflecting 5° from the first dorsal vertebra (overall 30° from the first caudal), a little different from the straight and slightly ventrally deflected posture previously described for other sauropods^9,15^. When using the lateral semicircular canals (LSCs) of the inner ear^25^ to reconstruct the position of the skull, it forms an angle of 10° with the neck, compatible with the non-deflected, osteologically induced curvature of the presacral vertebrae (Fig. 1).

When all scapular girdle bones remain in articulation and are placed in the ribcage following the results of independent osteological, myological and phylogenetic bracketing (see Methods and SI *appendix*, Section 5), the scapular girdle is mounted in the following way: sub-vertical scapulae, coracoids antero-ventral to ribcage, scapular heads anterior to the ribcage and glenoids a bit ventral to the distal tips of anterior dorsal ribs. This allows for an upright articulation of the humerus, as is known to be the condition for sauropods. The distal humerus to floor distance is 1.11 m.

The forearm and hand are not yet known in any *Spinophorosaurus* specimen. However, the aforementioned distance implies a forearm (0.748 m ulna) and hand (0.278 m metacarpal III + 0.06 m carpals) with similar proportions relative to the humerus to those of individuals of closely related non-neosauropod eusauropods (SI *appendix*, Section 4; Tables S1, S2). These reconstructed forearm and hand are, therefore, more parsimonious than assuming a proportionately longer or shorter forearm and/or hand.

Summing up, the osteologically induced curvature of the presacral column of *Spinophorosaurus* makes its skull deflect 30° dorsally from the first caudal vertebra in osteologically neutral posture, with the snout situated at about 5 m above the ground, more than twice as high as the shoulder and acetabulum, at 2.15 m (see Fig. 2A).

### *Spinophorosaurus* neck range of motion analysis

The neck of *Spinophorosaurus* has 12 moderately elongated cervical vertebrae (largest average Elongation Index, aEI = 3.63, see Supplementary Information Section 1 for details) with two features increasing range of motion in the dorsoventral plane. The prezygapophyseal facets on the cervical vertebrae are particularly antero-posteriorly elongated next to those of other sauropod cervical vertebrae (Fig. S5A). These elongated articulation facets provide a greater range of motion per joint than in other sauropods in comparable joint positions (Fig. S5C, D). The 12^th^ cervical vertebra has all traits expected of a cervical, including a short and small cervical rib. The first dorsal vertebra of *Spinophorosaurus* shows a tendency to cervicalization, but it already bears a dorsal rib (Fig. 1E). This vertebra is more elongated than any of the other dorsal vertebrae and its centrum has a trapezoidal shape in lateral view (its anterior condyle is more dorsally located than the cotyle). Also, the prezygapophyseal rami are anteriorly projected, the prezygapophyseal facets have the same elongation present in the preceding cervical vertebra and the ventral keel is very pronounced. Most of these characters are missing in the second dorsal vertebra (Table S3). This partial cervicalization makes the first dorsal vertebra a functional cervicodorsal vertebra, sharing many convergences with the cervicalized first thoracic vertebra of giraffes: longer than wide prezygapophyseal facets, a ventral keel in the centrum or a centrum length intermediate between that of the preceding cervical and the following dorsal vertebra (Fig. S6, Table S3). Despite having a dorsal rib, the cervicothoracic vertebra of giraffes has a greater range of motion than any of the other thoracic vertebrae, but more reduced than the other cervical vertebrae^26^. This situation is also present in *Spinophorosaurus*, in which the first dorsal vertebra has an amount of osteological mobility greater than that of the following dorsal vertebrae but smaller than that of the preceding cervical vertebra (Table 2). This is particularly noticeable on its first dorsal vertebra and, to a lesser degree, on the second and third because their prezygapophyseal facets and centra are shorter than on the first dorsal.

**Table 2:** Dorso-ventral range of motion values at the cervical-dorsal boundary for *Spinophorosaurus nigerensis*, *Plateosaurus engledhardti, Okapia johnstoni* and *Giraffa camelopardalis*, measured as degrees between maximum dorsiflexion and ventriflexion in the referred vertebra.

	DV 3	DV 2	DV 1	LCV	PCV
*Okapia*	3	4	3	9	17.5
*Giraffa*	5	4	14	25–27	25–28
*Plateosaurus*	4	4	8	10	10
*Spinophorosaurus*	*7	*9	17	27	28

DV = Dorsal Vertebra; LCV = Last Cervical Vertebra; PCV = Penultimate Cervical Vertebra. *Asterisk indicates estimated measurements based on field pose (dorsiflexion) and osteological stop of ribs (ventriflexion). Data from *Okapia* and *Giraffa* from Gunji & Endo^26^. Data from *Plateosaurus* from Mallison^52^.

The range of motion of the neck alone (without deflecting the first dorsal from ONP) allows placing it vertical, with the skull antero-posterior axis perpendicular to the ground without disarticulation (Fig. 2A). The inner ear semicircular canals are relatively very large and slender^25^. This has been related with the perception of angular accelerations of the head of animals, predicting a highly flexible neck for *Spinophorosaurus*^25^, corroborated at least in the dorso-ventral plane by our range of motion analysis (Fig. 2A).

## Discussion

### Body plan and feeding capabilities of *Spinophorosaurus*

Although there has been a thorough debate on the feeding capabilities of different sauropod taxa, most analyses have focused on skull, teeth and neck functional morphology. Previous studies showed that pectoral girdle and forelimb position created a dorsal sloping of the presacral vertebrae^14,20^, suggesting that studying only cranial and cervical anatomy might be insufficient to understand sauropod body plan and feeding capabilities^14^. Mounting the virtual skeleton of *Spinophorosaurus* has revealed that the morphology of sacrum and posterior dorsal vertebrae also have a crucial role on the overall body plan by deflecting the presacral series from the caudal series in osteologically neutral pose (Fig. 1, Fig. 4S). Given the general position of the pelvis in Sauropoda, with an antero-posteriorly projected ilium and a mesopubic and opisthopubic condition^24^, the coalesced sacrum is situated so that the posterior face of the last sacral centrum is sub-vertical. This makes the presacral series to slope dorsally and the tail to be subhorizontal (Figs. 1 and 4S). Since a subhorizontal tail has been known to be present in the majority of known sauropods^27–29^, the OIC of the tail of *Spinophorosaurus* is therefore compatible with this condition.

The dorsal sloping of presacral vertebrae in sauropod dinosaurs was noticed by Gilmore when assembling *Diplodocus sp*. USNM 10865, in which the posteriormost dorsal vertebrae would dorsally deflect from the sacrum when in articulation^27^. Gilmore also noticed that the posteriormost dorsal vertebrae of USNM 10865 had an anteriorly pointed neural spine which was perpendicular to the ground when vertebrae were in articulation with one-another and with the sacrum^27^. The neural spines of posterior dorsal vertebrae DV12 to DV9 are also anteriorly directed in the holotype of *Spinophorosaurus* (Fig. 1). However, Gilmore remarked that around mid-thoracic region, the dorsal vertebrae series of USNM 10865 reversed its curvature, deflecting ventrally, resulting in an arched torso^27^. This arching of the torso is not present in *Spinophorosaurus* in osteologically neutral pose, nor there is any wedging in middle or anterior dorsal vertebrae to create a ventral deflection (Figs. 1, S4). Instead, the presacral column of *Spinophorosaurus* is very straight, with only a slight dorsal deflection of 10° from the last dorsal vertebra to the axis. This configuration of a dorsally sloping presacral column in *Spinophorosaurus* is compatible with the sub-vertical, more antero-ventrally placed scapulocoracoid proposed by independent osteological^20,30^, myological^31^, biomechanical^32^ and phylogenetical bracketing^20^ studies (see Methods and SI *appendix*, Section 5), as well as with an elongated scapula and humerus.

Forelimb length and shoulder height are important factors for estimating the feeding capabilities of sauropod dinosaurs. Those with longer forelimbs relative to their hindlimbs are interpreted as having high browsing capabilities^14,15^. *Camarasaurus* is a genus typically interpreted as a capable medium to high browser based on its shoulder height^14,33^, with a humerus to scapula ratio around 0.74 and a femur to humerus ratio around 1.37. Both the humerus to scapula ratio (0.816) and the estimated femur to humerus ratio (1.21) of *Spinophorosaurus* indicate a relatively larger humerus than that of *Camarasaurus*. The scapula of *Spinophorosaurus* is also slightly longer than its femur, whereas that of *Camarasaurus* is slightly shorter than its femur (Table 1). Therefore the preserved forelimb and pectoral girdle elements of *Spinophorosaurus* are relatively longer than those of *Camarasaurus*. All in all, *Spinophorosaurus* had a humerus and scapula relatively longer, making its shoulders relatively taller than those of most known sauropods, with the exception of at least *Atlasaurus*^34^ brachiosaurids^10,33,35^ and some titanosaurs (Table 1, Fig. 2B).

Regarding the missing forearm and hand bones in *Spinophorosaurus*, if they were as long relative to the humerus as in other non-neosauropod eusauropods (Table S1), they would be compatible with those in the virtual mount. Shorter or taller shoulder height would require, respectively, shorter or longer hypothetical forearm and hand relative to the humerus. This implies that any hypothesis regarding much shorter or much taller shoulders for *Spinophorosaurus* than those proposed in this reconstruction requires additional evolutionary steps (to acquire relatively shorter or longer missing elements), and are therefore less parsimonious.

This shows that the overall body plan of this sauropod has a clear tendency toward vertical lengthening due to its sacral wedging as well as its humerus to femur and scapula to femur proportions, especially when compared with its earlier branching relatives (Table 1). This tendency to verticalization, coupled with the increased dorso-ventral neck flexibility granted by the elongated prezygapophyseal facets, and coordinated by its relatively large and slender inner ear^25^, reveals a feeding envelope with a large vertical component. This feeding envelope is greater than those calculated for *Diplodocus carnegii*^9^, *Apatosaurus louisae*^9^ or *Mamenchisaurus youngi*^6^, and possibly greater than those of *Camarasaurus* or *Haplocanthosaurus* (SI *appendix*, Section 8; Fig. S4). Summing up, *Spinophorosaurus* can be interpreted as adept at high browsing, like modern giraffes are^36^: its skeleton conferred *Spinophorosaurus* the capability to browse on vegetation at nearly three times the height of its shoulders, and hence it might have been part of its feeding strategy.

### Sauropod sacra as an evolutionary innovation

Given the high disparity of hindlimb/forelimb length proportions among Sauropoda^37,38^, the evidence of different feeding capabilities among different sauropods^3,11,14^ and the role of the wedged sacrum on the high browsing capabilities in *Spinophorosaurus* (due its high impact on vertebral column OIC), a hypothesis can be formulated: only sauropod dinosaurs with moderately to extremely elongated forelimbs would have strongly acute wedged sacra and dorsal vertebrae.

**Figure 3:**
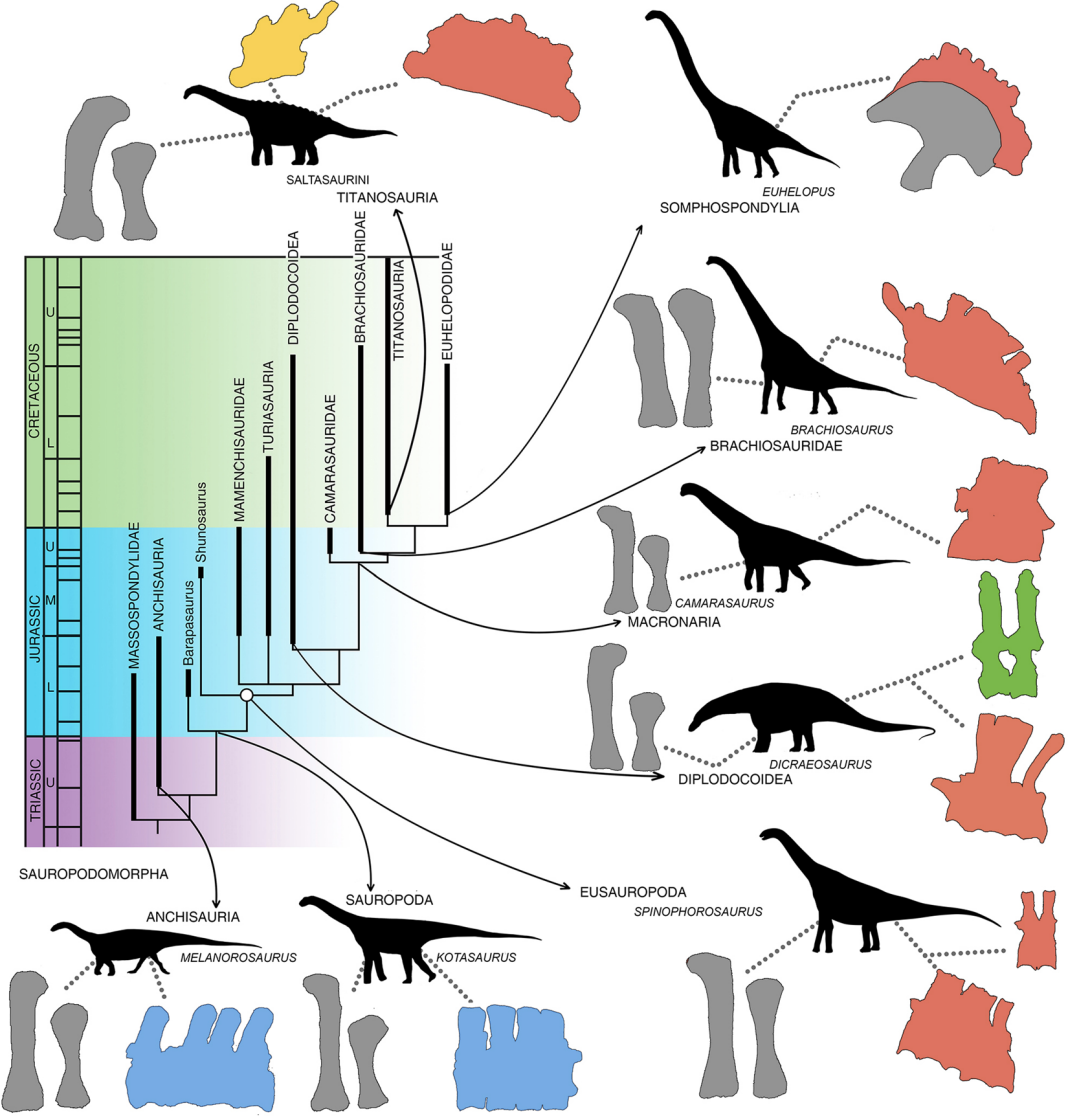
Time calibrated sauropodomorph phylogenetic relationships with emphasis on different body proportioned taxa. Different body proportions for sauropodomorph dinosaurs include (1) facultative quadrupedalism and medium height browsing in non-sauropod sauropodomorphs (e.g. *Melanorosaurus*) with non-wedged sacrum; (2) obligatory quadrupedalism and medium height browsing in basally branching sauropods with longer forelimbs but no wedged sacrum (e.g. *Kotasaurus*); (3) medium-high browsing in non-neosauropod eusauropods, with longer necks and forelimbs and an acute wedged sacrum (e.g. *Spinophorosaurus*); (4) medium-ground level browsing in dicraeosaurid and rebbachisaurid diplodocoid sauropods, with short necks, shorter forelimbs than non-neosauropod sauropodomorphs but with acute wedged sacrum and obtuse wedged dorsal vertebrae (e.g. *Dicraeosaurus*); (5) medium-height browsing in basally branching macronarian sauropods, with a wedge shaped sacrum and retroverted pelvis (e.g. *Camarasaurus*); (6) extreme high browsing in brachiosaurid sauropods, with extremely elongated necks, humeri longer than femora and extremely wedged sacra (e.g. *Brachiosaurus*); (7) extreme high browsing in euhelopodid titanosauriforms, with extremely long necks and extremely wedged sacra (e.g. *Euhelopus*); and (8) medium-low browsing in some lithostrotian titanosaurs, with shorter forelimbs than other titanosaurs and titanosauriforms but still retaining a wedged sacrum and with obtuse wedged dorsal vertebrae (e.g. Saltasaurini). A wedged sacrum is only found in Eusauropoda, and albeit the degree of wedging varies among sauropods, it never returns to the basal condition. Schematic bones obtained directly from digitized 3D models, except in *Melanorosaurus*^53^, *Kotasaurus*^39^, *Dicraeosaurus*^54,55^ and *Euhelopus*^56^, where photographs were used as reference. Femora and humeri are proportioned to each other following Table 1. Schematic bone color coding: rectangular sacra (blue); acute wedged sacra (red); obtuse wedged mid-dorsal vertebrae (yellow and green). Time callibration of nodes after Xu *et al*.^47^.

Interestingly, a comparison of sacral wedging with the relative lengths of humeri and femora reveals a correlation: lower femur maximum length/humerus maximum length ratios are associated with a higher wedging angle of the sacrum and vice versa (Fig. 2B). However, all eusauropod sauropods have wedged sacra, even those with shorter humeri (Figs. 2B, 3). Sauropods with extremely short humeri (dicraeosaurids and some titanosaurs) have sacrum wedging angles of around 10° or a little higher, whereas extremely tall-shouldered sauropods with humeri longer than femora (Brachiosauridae) have sacrum angles of up to 30° (Table 1). This sacrum wedging to humerus relative length correlation, however, is only found in Eusauropoda. There are few known non-eusauropod sauropods preserving sacral and limb material, but those which preserve complete sacra have very little to no wedging (i.e. *Kotasaurus*^39^ or *Barapasaurus*^40^) despite having columnar fore and hindlimbs (Fig. 3) and being obligatory quadrupeds. The sacrum in non-sauropod sauropodomorphs is also hardly wedged even in large, quadrupedal species (i.e. *Melanorosaurus*^41^). Some of these quadrupedal non-sauropod sauropodomorphs have relatively larger humeri than some eusauropods, that also were obligatory quadrupeds but had wedged sacra (i.e. *Shunosaurus*^42^). Therefore, a rectangular or barely wedged sacrum would be the basal condition in Sauropodomorpha, which eventually derived to a more strongly wedged one in Eusauropoda, 10° or more (Fig. 3). From this point on, the relative length of the humerus correlates with the amount of wedging in the sacrum, which does not happen in non-eusauropod  sauropods (Fig. 2B).

This correlation between the sacrum and forelimbs appears to have worked as a functional module during sauropod evolution, with evolutionary changes in limb proportions and sacra happening in a reciprocal way (Fig. 2B): changes in limb proportions resulted in modifications on the angulation of the sacrum wedging. In those sauropods with extremely short forelimbs and low/ground level browsing capabilities, the sacral wedging diminishes, making the presacral series deflect dorsally with a lesser angle. The sacrum, however, never returned to the basal condition of a more rectangular sacrum (Fig. 2B, Table 1). The acute sacrum wedging in sauropods with extremely short forelimbs, such as *Dicraeosaurus*, is also counteracted by an obtuse wedging in the dorsal vertebrae, which makes the presacral vertebral series deflect ventrally (Fig. 3, Figure 6.5C in Stevens and Parrish^15^). Some of these sauropods with shorter humeri had proportionately shorter necks than other sauropods^43^, and medium-height browsing^44,45^ or ground level browsing^3,45^ capabilities have been proposed for them. Some titanosaurs with relatively shorter necks and forelimbs might also have been medium to ground level browsers, and they also have wedged sacra and obtuse wedging in the dorsal vertebrae (Fig. 3, Saltasaurini), although more functional analyses are necessary. Sauropods with shorter forelimbs arose separately in at least two different clades according to phylogenetic analyses^38,46–48^ (Fig. 3, Diplodocoidea and Titanosauria) and all of them had close relatives with longer forelimbs and more wedged sacra. This implies that the acute wedged sacrum became irreversible for Eusauropoda, thus fixed in their body plan, likely a constraint for sauropods that evolved lower browsing feeding strategies.

The evolution of an acute wedged sacrum in sauropods appears to have been abrupt (Fig. 3), turning the non-wedged sacrum of basally branching Early to Middle Jurassic sauropods such as *Barapasaurus* and *Kotasaurus*, into a quite acute wedged one in Middle Jurassic sauropods such as *Shunosaurus*^42^ or *Patagosaurus*^49^. This derived sacrum evolved well after sauropods had already become obligate quadrupeds, earlier in their evolution^46^, implying it was not linked to the evolution of quadrupedality. Neck elongation has typically been regarded as one of the most important key innovations in sauropod evolution^48^, directly affecting the size of the feeding envelope^1^. Feeding envelope size, and with it, feeding efficiency, increased primarily from neck elongation^8^. However, the appearance of strongly wedged sacral and dorsal vertebrae and changes in limb proportions might also have been important factors on feeding envelope size. As evidenced for the first time in the almost complete skeleton of *Spinophorosaurus* (Figs. 1, 2B), further increase in the vertical component of feeding envelopes could have been achieved by relative forelimb elongation, longer prezygapophyseal facets in cervical vertebrae, cervicalization of anterior dorsals, and an acute wedged sacrum, the latter of which occurred in sauropods more deeply nested than *Kotasaurus* and *Barapasaurus* (Fig. 3).

The fact that an acute wedged sacrum of more than 10° remained present in all eusauropods, supports the trait as a synapomorphy rather than a convergent character (Fig. 3). Moreover, it may represent a new factor in the evolutionary cascade proposed for sauropod gigantism^1,8^, directly affecting the energy-efficient feeding selective advantage proposed in that evolutionary cascade.

## Conclusions

**Figure 4:**
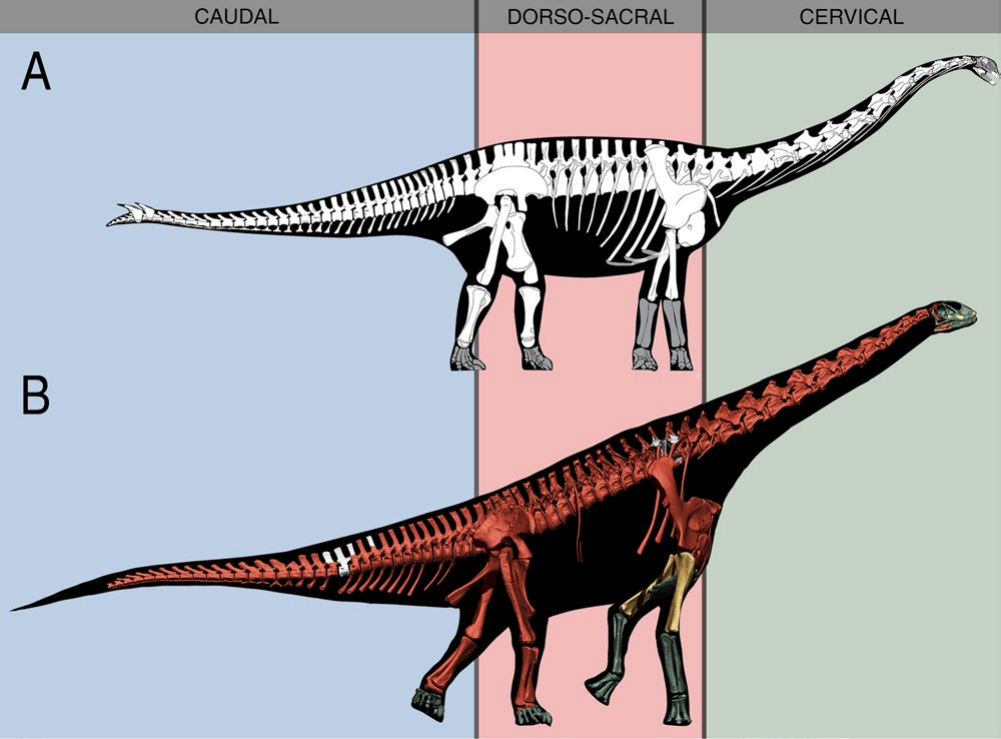
Skeletal reconstructions of *Spinophorosaurus nigerensis*. (**A**) Original skeletal reconstruction of *Spinophorosaurus* first published in 2009, modified from the original publication^21^. (**B**) Virtual skeletal reconstruction of *Spinophorosaurus*. All the skeletal reconstructions have been scaled to the same dorso-sacral sector length (between the last sacral vertebra and the first dorsal vertebra). Previous reconstruction lacked information on precise sacrum morphology, hence the radically different osteologically induced curvatures of the presacral vertebrae. Other bones which differ between the 2009 reconstruction and actual fossils (as further preparation has been carried out) are the ilium, the first three dorsal ribs, the cervicodorsal transition and the anterior caudal vertebrae.

The virtual skeleton of *Spinophorosaurus nigerensis*, the first digital mount using a single specimen from a sauropod dinosaur so complete, reveals a body plan very different from previous reconstructions of this animal, with the presacral column antero-dorsally deflected and relatively tall shoulders (Fig. 4). A 20° wedged sacrum in lateral view (Fig. 1) causes most of this dorsal sloping of the presacral column. Elongated pre and postzygapophyses and a partially cervicalized first dorsal vertebra enabled a greater dorso-ventral range of motion in *Spinophorosaurus* than in all previously studied sauropods. This enlarged vertical component of the feeding envelope, together with the dorsal sloping, show *Spinophorosaurus* had high browsing capabilities. Thus, skeletal adaptations to high browsing in sauropods were present as early as the Early-Middle Jurassic (Fig. 3).

All eusauropods shared a sacrum wedged more than 10° inherited from a common ancestor, which evolved in correlation with humerus/femur proportions in what could be part of a functional module: the greater the sacral wedging, the longer the humerus relative to the femur. This skeletal adaptation was likely a synapomorphy and a key factor on the evolutionary cascade proposed for sauropod gigantism and for the evolution of their body plan, as it remained present in all known eusauropods until their extinction at the K-Pg boundary.

## Material and Methods

### ***Spinophorosaurus*digitization**

The holotype (GCP-CV-4229 and NMB-1699-R) and paratype (NMB-1698-R), found next to the holotype^21^ (SI *appendix*, Section 2), were both scanned by digital photogrammetry, using the protocol described by Mallison and Wings^50^. As many bones were collected in multiple good-fitting fragments but were not put back together after preparation when the scans were performed, the fragments were photographed separately and put together in Agisoft Photoscan 1.3 using a virtual alignment technique (SI *appendix*, Section 3). Although the skeleton is exquisitely preserved with minor symmetrical latero-medial compression, two middle caudal vertebrae and dorsal vertebrae DV2 and DV3 were affected by a fault, and therefore had slight shear distortion and some breakages. They were digitally restored under the protocol proposed by Vidal and Díez Díaz^51^ (SI *appendix*, detailed in Sections 3 and 4).

### **Mounting the virtual*Spinophorosaurus*skeleton**

The skeleton was assembled in ZBrush 4R6 in osteologically neutral pose (ONP, see Terminology in SI *appendix*, Section 1), that is, maximum overlap of the pre- and postzygapophyseal facets. To reconstruct missing bones from the holotype specimen, the approach was a combination of scaling known elements from the paratype and phylogenetic interpolation for elements unknown in both specimens. Broken dorsal vertebrae DV2 and DV3 and caudal vertebra CdV15 were reconstructed using field photographs as reference, as well as the immediately anterior or posterior elements with good preservation (SI *appendix*, detailed in Section 4). The humerus to scapula maximum length ratio was measured in the paratype specimen of *Spinophorosaurus* as 0.816. The missing humerus of the holotype was therefore estimated to be 1014 mm long (0.816 times the 1243 mm scapula). The missing forelimb elements (ulna, radius and hand) were estimated based on known forelimb proportions of closely related non-neosauropod eusauropods (Table S3). The final model has a 748 mm ulna and a 278 mm longest metacarpal, both closer to the shorter end of the spectrum of the estimated lengths (Table S2) and compatible with the skeletal reconstruction proposed (SI *appendix*, detailed in Section 4 and Tables S2, S3). Axial elements were assembled following the protocol of Mallison^52^ (SI *appendix*, Section 5) in which vertebrae were articulated in pairs (only two elements visible at once, one remained static while the other was articulated in ONP) in order to minimize preconceived notions on axial skeleton “neutral” posture, maximizing an axial skeleton curvature based on bone geometry. It was done anterior to posterior and vice versa. If both skeletal assemblages had the same osteologically induced curvature (OIC), the mount was approved for further work.

The pelvic girdle was posed with an antero-posteriorly oriented ilium and a mesopubic condition, the widespread condition for all sauropods^24^. The pectoral girdle was mounted with sub-vertical scapulae with the coracoids antero-ventral to the ribcage, the scapular heads anterior to the ribcage and the glenoids a bit lower than the distalmost tips of the anterior dorsal ribs (Fig. 1). This position and orientation of the pectoral girdle is the only possible position which allows (i) to keep the scapulocoracoids articulated with the clavicles and interclavicle^30^ and fit within the ribcage, (ii) to not have the ribcage become an osteological stop for humerus retraction, (iii) to have functional cingulo-axial and shoulder musculature lines, particularly for *M. subcoracoscapularis pars scapularis*, which has its origin on the acromial region, on the medial side^31,32^ and which would otherwise be obstructed by the ribs, (iv) to place the costo-coracoideal articulation subparallel to the distal ribs axis as is the case of all extant non-mammalian tetrapods^20^, (v) to leave room dorsal to the distal expansion for the cartilaginous suprascapula, which would be the insertion point for *M. rhomboideus*^31,32^ (SI *appendix*, detailed in Section 5). This configuration of the scapulocoracoid has been also been independently reconstructed on previous works^20,31,32^. The appendicular skeleton was articulated in ONP, then posed in a fast walking gait for the figures.

### Range of motion analysis

While there are standards for comparing skeletal neutral postures, there are no standards defined yet for range of motion analyses: different authors follow different criteria for assessing maximum articular excursions (SI *appendix*, Section 6). Here, the protocol of Mallison^52^ was used: vertebrae were deflected until only a minimum overlap of the facets was retained, that is, before they completely disarticulated (Fig. S5). That way, if accounting for a larger facet *in vivo* (as happens in extant archosaurs^52^), the range of motion is underestimated rather than overestimated. The center of rotation was the anteriormost part of the condyle of the posterior vertebra when the articulation was opisthocoelus, and at midheight of the posterior centrum face in platycoelus articulations. This would prevent the misalignment of the neural canals (SI *appendix*, detailed in Section 6). When discussing browsing heights (low/medium/high browsing) we are not referring to behavior/paleobiology, but to the physical capabilities the osteological range of motion enabled (See Terminology in *SI*).

## Supplementary information

Supplementary Information.

Supplementary Video 1.

Supplementary Table 1.

## Data Availability

Most fossil material for the holotype specimen of *Spinophorosaurus nigerensis* (GCP-CV-4229) is temporally deposited at the Museo Paleontológico de Elche (Elche, Spain). The paratype specimen (NMB-1699-R) and part of the holotype (NMB-1698-R) of *Spinophorosaurus nigerensis* are temporally deposited at the Staatliches Naturhistorisches Museum (Braunchsweig, Germany). Both specimens will eventually be returned to the Musée National Boubou Hama (Niamey, Niger). The digital fossils used to build the virtual skeleton are deposited and accessioned at the Museo Paleontológico de Elche.
