# Supplementary material for: High browsing skeletal adaptations in Spinophorosaurus reveal an evolutionary innovation in sauropod dinosaurs
Source: Sci Rep. 2020 Apr 20;10:6638. doi: 10.1038/s41598-020-63439-0 (PMC7171156; doi:10.1038/s41598-020-63439-0)
Supplement: Supplementary file 1 — Supplementary Information. [file 41598_2020_63439_MOESM1_ESM.pdf]

Supplementary Information for

High browsing skeletal adaptations in *Spinophorosaurus* reveal an evolutionary innovation in sauropod dinosaurs

Authors: D. Vidal[1*], P. Mocho[2,1,3], A. Aberasturi[4], J.L. Sanz[5,6], F. Ortega[1]

Affiliations:

[1]Grupo de Biología Evolutiva, Facultad de Ciencias, UNED, Paseo Senda Del Rey, 9, 28040 Madrid, Spain.

[2]Instituto Dom Luiz, Universidade de Lisboa, Bloco C6, 38Piso, sala 6.3.57, Campo Grande, 1749-016 Lisbon, Portugal.

[3]The Dinosaur Institute, Natural History Museum of Los Angeles County, 900 Exposition Blvd., 90007 CA, Los Angeles, USA.

[4]Museo Paleontológico de Elche, Carrer Sant Joan, 3, 03203 Elche, Spain.

[5]Unidad de Paleontología, Facultad de Ciencias, Universidad Autónoma de Madrid, Calle Darwin, 2, 28040 Madrid, Spain.

[6]Real Academia Española de Ciencias Exactas, Físicas y Naturales, Calle Valverde, 24, 28004 Madrid, Spain.

*Correspondence to: eoalulavis@gmail.com.

This PDF file includes:

Supplementary Text
Figs. S1 to S6
Tables S1 to S3
References for SI reference citations

Supplementary Text

1. **Terminology**
    a. **Browsing height**
        i. **Ground-level browsing**
        ii. **Low browsing**
        iii. **Medium-height browsing**
        iv. **High browsing**
    b. **Cervicalization**
    c. **Elongation Index (EI)**
    d. **Evolutionary Innovation vs Evolutionary novelty**
    e. **Neutral Bone Only Posture (NBOP)**
    f. **Osteologically Induced Curvature (OIC)**
    g. **Osteologically Neutral Pose (ONP)**
        i. **Intervertebral ONP**
        ii. **Appendicular joints ONP**
    h. **Osteological Stops**
    i. **Retroverted pelvis**
    j. **Wedged Vertebrae**
2. ***Spinophorosaurus nigerensis* specimens**
3. **Digitizing the bones of *Spinophorosaurus nigerensis***
    a. Holotype digitization
    b. Retrodeformation
    c. Digital separation of elements

   d. **Skull**

4. **Reconstruction of missing elements**

   a. **Forelimb**

   b. **Foot**

5. **Assembling the virtual *Spinophorosaurus nigerensis***

   a. **Axial skeleton**

   b. **Girdles and appendicular skeleton**

   c. **Physical model articulation**

6. **Cervical range of motion analysis in *Spinophorosaurus nigerensis***

   a. **Cervical Ribs**

   b. **Measuring the range of motion**

7. **Morphofunctional convergence at the cervicodorsal boundary between *Spinophorosaurus nigerensis* and *Giraffa camelopardalis***

8. **Cervical range of motion in other sauropods**

9. **Wedged sacra and forelimb length in sauropods**

   a. ***Diplodocus***

   b. **Brachiosauridae**

   c. ***Mamenchisaurus youngi***

   d. ***Dicraeosaurus***

1. Terminology

The terminology of this paper follows that of previous authors, although some minor adjustments have been made to narrow the precision of the terms employed. The implications of the different definitions given to some terms are further discussed in order to remain as precise as possible:

- **Browsing height:** there have been four categories described for interpreting the height at which sauropod dinosaurs fed, namely:

  Ground-level browsing: feeding at ground level[1].

  Low browsing: feeding with the head below shoulder height / cervicodorsal transition height[2].

  Medium-height browsing: feeding with the head kept between shoulder height and half neck length above the shoulders, about 30°[2].

  High browsing: feeding with the neck more inclined than 30°[2].

  Comment: Most of these categories have quite arbitrary limits, and most are not mutually exclusive, since a high browser (e.g. *Giraffa*) can browse lower than its shoulder height. These terms refer to the capabilities of an organism, not to their actual role in an ecosystem (an small sized organism may be able to browse with the neck more inclined more than 30° but won't physically reach the higher vegetation canopy). Therefore, these terms should be used as descriptive on the physical capabilities of fossil organisms rather than on their behavior or ecological role.

- **Cartilaginous Neutral Pose (CNP):** the term was coined by Taylor[3] for "the pose found when intervertebral cartilage [that separates the centra of adjacent vertebrae] is included". Since the amount of inter-vertebral space cannot be certainly known for most fossil vertebrate taxa, true CNP will likely remain unknown for most taxa or always based on estimates.
- **Cervicalization:** the term is usually employed as the incorporation of dorsal vertebrae into the neck[4] while maintaining the same number of presacral vertebrae as ancestors without cervicalized dorsals. **The first dorsal vertebra acquires the characters of a cervical vertebra** (i.e. centra as long as tall or longer than tall, prezygapophyses projected anteriorly to the anterior face of the centrum, longer prezygapophyseal facets). **Those characters enable it to become functionally part of the neck.** Cervicalization is a complex process with several homeotic genes involved, and not all the characters may appear at once[5]. Therefore, when anteriormost dorsal vertebrae have both cervical and dorsal characters in mosaic fashion, the term **partial cervicalization** is employed herein.

Elongation Index (EI): it was devised by Upchurch[6] as a way to quantify the elongation of a vertebral centrum, *"the length of a vertebral centrum divided by the width across its caudal face"*. Wilson and Sereno[7] used the height of the centrum instead of the width, and Wedel et al.[8,9] used the latter definition with the former name "elongation index". Since taphonomic deformation can drastically alter the shape of a vertebral centrum, using width or height may render different values for the same vertebra, Chure et al.[10] coined "aEI" to

the *"scale [of] centrum length to the average of centrum height and width (aEI) to avoid confusing changes in centrum elongation with those in cross-sectional shape as well as to account for deformation"*, which is the index followed in this publication.

- **Evolutionary Innovation vs Evolutionary novelty**: Erwin[11,12] differentiates evolutionary innovation from evolutionary novelty. An evolutionary novelty is when a newly individuated character or feature of an organism not present in an ancestral species appears but does not necessarily have an impact in diversity or disparity of the lineage on which it appears. An evolutionary innovation is when whether a newly individuated character appears or a previously present feature derives into a new character state and it has a noticeable impact of the diversity or disparity of the lineage.
- **Neutral Bone Only Posture (NBOP):** Paul[13] coined this term for the definition of "neutral deflection" given by Stevens and Parrish[14], including zygapophyseal alignment and vertebral centra alignment criteria (contra ONP, which would only account for the zygapophyseal alignment criterion, see below). The definition of Steven and Parrish is exhaustive and very complete: "*Posture of an intervertebral joint defined by the full articulation of the zygapophyseal facets, with platycoelous vertebral centra surfaces or the rims of cotyle-condyle (procoelous or opisthocoelous) articulations parallel*". ONP may or may not coincide with NBOP (Fig S1).

- **Osteologically Induced Curvature (OIC):** Stevens[15] defines it as *"the curvature of a vertebral column in ONP, as distinguished from curvature induced by joint deflection".*
- **Osteologically Neutral Pose (ONP)**:

  General definition: *Maximum* alignment of articular surfaces.

  Synonyms: neutral pose[16], neutral deflection[14,17], zygapophyseal alignment[18], optimal fit, best fit[2] neutral articulation[13].

  Specific definitions: This term is common in literature from the last decades to present, and seeks to find the pose achieved by the maximum osteological articulation of a skeleton. However, precise definitions vary slightly depending upon the anatomical region referred (axial and appendicular definitions necessarily vary) and sometimes authors have given slightly different definitions and different names to the same concept, rendering its usage a bit imprecise. Despite this, there appears to be an underlying consensus about its usage as the maximum overlap of articular surfaces. More specific and exhaustive definitions for the different types of bones can be given after reviewing previous works:

  Intervertebral ONP: Stevens and Parrish first used the concept just as "neutral pose" in their 1999 landmark paper, defining it as *"wherein the paired articular facets of the postzygapophyses of each vertebra were centered over the facets of the prezygapophyses of its caudally adjacent counterparts"*[16]. Their study centered on the axial skeleton and the

zygapophyseal facets in particular, and therefore this first definition was in accordance with the variables that these authors explored. Nevertheless, it is the most extensively used definition for osteological neutral pose (and synonyms) of the axial skeleton, with the same general concept used a few years later by Stevens: (as neutral deflection) *"the zygapophyses* [were] *centered post above prezygapophyses"*[17] and (as osteological neutral pose) *"centering the associated pre- and postzygapophyses"*[15]. Other authors have also used the maximum zygapophyseal facets overlap as the criterion defining ONP. Christian and Dzemski[19] positioned *"the centres of the facets of the postzygapophyses above the centres of the prezygapophyses of the caudally adjacent vertebrae"* in extant vertebrates. Taylor, Wedel and Naish[20] used *"maximum overlap between the zygapophyses",* as well as Mallison[21,22] *"maximal overlap of the zygapophyses".* Some authors have also implemented different criteria of the vertebral centra in their definitions of ONP as well as the maximum overlap of the zygapophyses. Christian[2] considered ONP *"bringing post and prezygapophyses of adjacent vertebrae into contact, so that the joint between the centra was articulated and the joint facets of pre and postzygapophyses were centered above each other".* Stevens and Parrish[14] used a more precise criterion for the articulation of centra, coining neutral deflection as *"defined geometrically by the alignment of the zygapophyses and by nulling the deflection at the central articulation. Pre and postzygapophyses are centered within their range of dorsoventral travel when the two vertebrae are in their undeflected state. Simultaneously,*

the central facets will be in a neutral or undeflected state. For platycoelous vertebrae, the two planar articular surfaces are parallel when undeflected. Determining neutral position for opisthocoelous vertebrae requires closer scrutiny of the margins of the central articulation. The synovial capsule surrounding the condyle-cotyle exhibits circumferential attachment scars. These ridges are parallel when the joint is undeflected". Carabajal et al.[23] used the same concept in their work, *"when the zygapophyses of successive vertebrae are aligned and the deflection at the central articulation is minimal"*. Paul[13] would coin the term "Neutral Bone Only Posture" for the pose in which *"the zygapophyses are in full, 100% overlapping articulation and the centra rims are parallel to one another"*.

There is an important caveat when trying to align the zygapophyses and set the centra rims parallel when using physical or virtual three dimensional vertebrae, which has been encountered thorough this work with opisthocoelous sauropod dinosaurs (Fig. S1) as well as with platycoelous modern human vertebrae[24]. When the zygapophyses are in 100% overlap (in all three dimensions, lateral-medial, antero-posterior and dorso-ventral planes) the centra rims are not parallel in most cases (Fig. S1). Studies on inter-vertebral cartilage have revealed that its thickness may be vary between different species, between individuals of the same species, particularly thorough ontogeny[25], or even within a single individual, depending on the region of the axial skeleton[25]. The zygapophyseal capsules, however, are not

thicker than a flat sheet covering[25], albeit enlarging the actual area of the articular facet outline (see below Range of Motion[3]). The preferred usage of the term ONP should refer only to the zygapophyseal joints, given i) the discrepancy between zygapophyseal and centra alignments which sometimes makes impossible to align both at once, ii) the more widespread use of the maximal zygapophyseal overlap criterion alone without the parallel centra criterion, and iii) the fact that intervertebral soft tissue thickness is more variable than zygapophyseal capsule thickness. The term “Neutral Bone Only Posture” coined by Paul should be used to include the centra rim criterion. Therefore, we propose the following definition for axial skeleton ONP in order to summarize exhaustively all previous definitions: *Posture of an intervertebral joint defined by the full articulation of the zygapophyseal facets, with complete overlap of the facet surfaces in all three anatomical planes* (antero-posterior, lateral-medial, dorso-ventral; Fig S1A).

Appendicular joints ONP: The only definition stems from Reiss and Mallison’s work on the hand of *Plateosaurus*[26], where they defined it as *"the position in which the long axes of two bones articulating with each other are approximately parallel to each other in lateral view"*.

Osteologically neutral pose should be always taken explicitly as a consensual “standard” for comparing the intrinsic deflection of joints between different specimens and subsequent biomechanical analyses. Using the same “standard”

starting point, independent results from different researchers working on different specimens are therefore more easily comparable than when different starting points are used. However, the pitfall of taking morphofunctional and/or behavioral conclusions out of ONP alone must be avoided. ONP can be the source for morphofunctional and/or behavioral hypotheses that will have to be tested with additional evidence, but not the sole criterion for drawing conclusions on paleobiological hypotheses.

.

- **Osteological Stops**: Stevens[15] defines them as *"contact between vertebrae that limits angular deflection at a vertebral joint and provides load-bearing bracing against disarticulation"*. The term can be used in a broader sense for *any bone to bone contact which prevents further displacement of any given bone in the direction in which both bones collide*. The osteological stop may coincide with an *in vivo* stop (when soft tissue in the area of bone-bone contact would not be very extensive) or, more likely, may indicate that *in vivo*, extensive soft tissues might have stopped motion way before bones might have contacted.
- **Retroverted pelvis**: This term was coined by Paul[27] to describe a phenomenon first reported in the pelvis of *Cathetosaurus lewisii*[28], and later found to be widespread among other camarasaurids. This condition was described by Jensen as *"Ilia rotated around a transverse acetabular axis, lowering the anterior iliac processes approximately 0-20 degrees, ventrally, below the axis of the vertebral column"*[28]. He also suggested it conferred

Cathetosaurus mechanical advantages for bipedality. McIntosh et al[29] would later refer to this condition as *"the longitudinal axis of the ilium has been rotated counterclockwise by about 20º about a transverse axis through the acetabulum"* and suggest that not only other *Camarasaurus* specimens, but also other sauropods might have a similar condition.

Paul coined the term retroverted pelvis/hips[27] citing Jensen[28] for the definition and used it in subsequent works, giving a new, different take redefining retroverted pelvis as *"in which the dorsal series slopes dorsally relative to the horizontal axis of the entire pelvis"* and adding *"in some sauropods such as brachiosaurs pelvic retroversion results in the dorsal series sloping strongly up and forwards while the pelvis and tail remain horizontal relative to the ground, in other sauropods such as camarasaurs the dorsal-sacral-caudal series remains horizontal to the ground while the pelvis is rotated relative to the latter"*[13].

This latter redefinition of the term incorporates the wedging of the sacrum and its influence on the geometry of the axial skeleton. However, pelvic retroversion is a condition caused by the morphology of the ilia (particularly the pubic pedicle and the angle it forms with the antero-posterior axis of the ilium blade) and the orientation of the sacricostal yoke, independent from the wedging of the sacral vertebrae (Fig. S1 C and D). Therefore, we think the definition of "retroverted pelvis" is a character independent from the wedging of the sacrum, and it should specifically refer to the condition originally reported by Jensen and McIntosh: *Anteroventral rotation of about 20º of the*

ilium respect to the antero-posterior axis of the sacral vertebrae centra (Fig. S1D *contra* Fig. S1C).

- **Wedged Vertebrae**: The term describes *marked trapezoidal shape in the centrum of a platycoelous vertebrae in lateral view or in the rims of a condyle-cotyle (procoelous or opisthocoelous) centrum type.* If the wedging makes the ventral rim of the centrum longer than the dorsal rim, the adjacent vertebrae will form an **acute** angle with it in ONP, while a wedging with the ventral rim of the centrum shorter than the dorsal rim will create an **obtuse** angle between the adjacent vertebrae in ONP (Fig. S2).

2. *Spinophorosaurus nigerensis* specimens

Two specimens referred to *Spinophorosaurus nigerensis* have been retrieved to date. The holotype (GCP-CV-4229 and NMB-1699-R) is a nearly complete specimen consisting of a partial skull, an almost uninterrupted axial sequence spanning from the second cervical vertebra (axis) to the 31st caudal vertebra, all right-side ribs and most of the left ones and chevrons. The pectoral girdle is almost entirely represented, only missing one coracoid and the sternal plates (if they were ossified). No elements from the forelimb were retrieved. The pelvic girdle is almost completely represented, but some elements are damaged, as most of the left ilium and the distal left ischium. Only the right femur, tibia, fibula and astragalus were retrieved. The axial skeleton was found articulated, with girdles and limbs partially scattered (Fig. S3). Some axial skeleton elements where damaged by micro-faults, but their relative position was not very distorted. The general outline was preserved faithfully on the second dorsal (Fig. S3B) and a little more damaged in the case of the 15th caudal vertebra (Fig S3C), but with enough detail to allow a digital reconstruction minimizing speculation (see below 3, digitizing the bones of *Spinophorosaurus nigerensis*).

The paratype (NMB-1698-R) was found next to the holotype, on the same stratigraphic level. Overlapping bones between both specimens are almost undistinguishable[30]. It is less complete than the holotype, but preserves some elements not retrieved in the holotype specimen, such as the humerus and parts of the preorbital region of the skull. Overlapping elements with the holotype include several cervical

vertebrae, a right scapula, several dorsal vertebrae fragments, a set of right ribs, a partial ilium, and some caudal vertebrae, caudal neural arches and chevrons.

3. Digitizing the bones of *Spinophorosaurus nigerensis*

Holotype digitization

Most bones from the holotype specimen were digitized by digital photogrammetry, using the same protocol described by Mallison and Wings[31]. As many bones were collected in multiple good-fitting fragments but were not put back together after preparation when the scans were performed, the fragments were photographed separately and put together in Agisoft Photoscan 1.3 using a virtual alignment technique. This technique consisted in digitizing the fragments while fitting them physically first, creating a 3D model termed "aligner" on a separate “chunk”. Then, the different fragments of the same bone were digitized separately on different chunks. Finally, the "align chunks" option from the software was used to align the separate fragments with the "aligner". This way, fitting the different fragments together to create the complete bone would take less man-time (although it took a larger computation-time) and with a fraction of mm error in the alignments.

Retrodeformation

The holotype specimen of *Spinophorosaurus* is very well preserved, and deformation is almost always due to compressive forces acting on the latero-medial axis in a symmetrical way, something evidenced by breakages and the shape of the vertebral centra, which are taller than wide. A few bones have slight shear, asymmetric deformation, but for the most part they only suffer the aforementioned compression and breakage. A protocol for retro-deforming elements in axial elements was proposed by Vidal and Díez-Díaz[32] and has been followed here on ZBrush 4R6.

Digital separation of elements

Some skeletal elements which would have not been permanently fixed in life (co-adjacent vertebrae would have been mobile in life, chevrons and ribs would be independent from their respective vertebrae, etc.) have become so due to fossildiagenetic processes, rendering further physical preparation to separate them hazardous to the integrity of the fossils.

To digitally separate those elements on ZBrush, the mesh were duplicated (once per each part meant to be separated) and then sliced to separate the parts, thus generating a separate mesh for each virtual bone desired. After separation, non-visible surfaces (i.e., medial side of prezygapophyseal rami or ribs) were sculpted. The surfaces were reconstructed based on (ranging from more accurate to less): i) the actual geometry of the bone when the missing area was very small; ii) the most proximal anterior and/or posterior accessible preserved elements, on vertebrae and chevrons; iii) the counterlateral bone in paired elements (ribs, girdles and limbs); iv) overlapping elements on the paratype and; v) overlapping elements from close relatives (see Vidal and Díez-Díaz[32], and particularly figure 4 on that paper for reference and below for the details used for the specific elements used on the virtual *Spinophorosaurus* reconstruction).

Skull

Most elements from the posterior region of the skull are preserved in the holotype specimen, with some overlapping elements in the paratype skull. The height of the skull was determined measuring from the complete skull roof to the ventral articular surface of the quadrate (which is preserved completely) and its antero-posterior length was

determined as a combination of the preserved complete palatines and the minimum number of teeth present in the majority of non neosauropod complete maxillae and/or dentaries (*Shunosaurus*[33], *Mamenchisaurus* , *Jobaria*[34], *Turiasaurus*[35], *Patagosaurus*[36]) . The preorbital region in both specimens is not well known, and hence the reconstruction figured is hypothetical, very preliminar and serves only as a placeholder for the head of the animal.

4. Reconstruction of missing elements

In order to reconstruct missing bones from the holotype specimen, the approach was a combination of scaling known elements in the paratype and phylogenetic interpolation for elements unknown in both specimens.

Forelimb

The forelimb is completely unknown in the holotype specimen of *Spinophorosaurus nigerensis.* The paratype, however, preserved one phalanx and fragmented metacarpal, the humerus and the scapula. Since the scapulae of both holotype and paratype are complete and the greatest scapula length to greatest humerus length ratio appears to be characteristic at the genus level (Table S1), even maintaining similar proportions in different ontogenetic stages at least in *Camarasaurus* (Table S1), the holotype was assumed to have had the same humerus to scapula ratio as the paratype.

For both antebrachium bones (ulna and radius), unknown in both specimens, proportions between humerus and ulna were calculated for sauropod specimens (all summarized in Table S1) that preserved all the elements within the phylogenetic context of *Spinophorosaurus nigerensis*. The most parsimonious estimates for greatest ulna length to greatest humerus length ratio range from 0.7 to 0.8. That estimate for *Spinophorosaurus* implies the proportions between the ulna relative to the humerus were the same as in its closest relatives, non-neosauropod eusauropods which preserve forelimbs from a single specimen (Table S1).

Metacarpal length was established the same way, scaling the longest metacarpal with the ulna (Table S1). The most parsimonious estimates for greatest metacarpal length to greatest ulna length ratio for *S. nigerensis* range from 0.35 to 0.39. The estimated

lengths for the ulna, radius and longest metacarpal are in Table S2, but on the skeletal reconstruction the exact values were a 748 mm ulna and a 278 mm longest metacarpal, both closer to the shorter end of the spectrum of the estimated lengths (Table S2). These estimates therefore consider no abrupt changes in forelimb proportions occurred in *Spinophorosaurus* relative to closely related sauropods: it is a more parsimonious hypothesis than assuming a proportionately longer or shorter forearm and/or hand.

Foot

The astragalus from the holotype specimen is the only foot element preserved in *Spinophorosaurus*. Since most eusauropod feet phalanges are fairly similar, a generic pes (minus the astragalus) based upon *Turiasaurus*[37], *Shunosaurus*[38], *Cetiosauriscus*[39], and *Ferganasaurus*[40] was used as a placeholder.

5. Assembling the virtual *Spinophorosaurus nigerensis*

The postcranial skeleton of the holotype is nearly complete, and thus the assemblage of the axial skeleton, girdles and hindlimb (except for most of the pes) could be carried out with less uncertainty than skeletons assembled from bones stemming from multiple individuals. Interpolated elements with estimated proportions and size based (see above and table S1) were articulated unmodified, and no further size or proportion adjustments were needed. The skeleton was assembled in ZBrush 4R6 in osteological neutral pose (see Terminology above).

Axial skeleton

Axial elements were assembled following the protocol of Mallison[21] in which the spine was split in different sectors (caudal, sacral, dorsal and cervical) and each sector was assembled separately. The assemblage was performed from anteriormost element to posteriormost and vice versa. All sectors were articulated in pairs (only two elements visible at once, one remained static while the other was articulated in ONP) in order to minimize preconceived notions on axial skeleton geometry. If both skeletal assemblages (anterior to posterior and vice versa) had the same Osteologically Induced Curvature (OIC, *sensu* Stevens[15]) the osteological neutral pose for that sector was considered positive. Available dorsal and cervical ribs were articulated with their tubercula and capitula in maximum articulation with diapophyses and parapophyses respectively.

A final, complete assemblage was done following Carpenter, Madsen and Lewis[41] using the conventional sequence for mounting a physical skeleton: i) sacrum and pelvis; ii) caudal vertebrae; iii) presacral vertebrae and ribs; iv) limbs; v) skull; vi) chevrons.

The sacrum was situated so that the first caudal vertebra was parallel to the horizontal plane, and from there, sectors where articulated again by pairs (with only two elements visible at once). By positioning the first caudal with its neural canal at roughly 0º deflection allowed to have a reference point to measure axial skeleton deflection, analogous to an origin of coordinates. This enabled us to contrast the results with the assemblages obtained following the protocol of Mallison (devised for mounting virtual specimens). Both yielded almost the same results (with negligible differences of less than 1º in the reconstructed osteologically induced curvatures).

The osteologically induced curvature (OIC) of the axial skeleton is as follows (Fig S4):

I) Cervical vertebrae articulate almost straight, but with a slight sigmoidal dorsal deflection. The posterior half of the neck (CV12-CV6) describes a very straight yet slightly dorsally sloping posture, while the anterior half is more dorsally deflected at the CV6-CV5 and particularly CV5-CV4 joints than preceding joints. Finally, a ventral deflection occurs at cervical vertebrae CV3 and the axis. It is therefore, though slightly different, in accordance with previous results on sauropodomorph neck ONPs such as *Plateosaurus*[22], *Mamenchisaurus*[2], *Diplodocus, Apatosaurus, Camarasaurus* or *Giraffatitan*[15].

II) Dorsal vertebrae articulate as a straight line dorsally deflected about 5º due to the slight acute wedging of the posteriormost two dorsal vertebrae, with no evidence of an arched dorsal series often reconstructed for other sauropods.

III) Caudal vertebrae also describe a sigmoid curvature, more pronounced than that of the neck. A slight ventral deflection of CdV1-12, more pronounced at the anterior half, changing gradually to a very slightly dorsally deflected region from CdV13-27, to gradually shift again to a slight ventral deflection until the last preserved vertebrae. All in all, CdV31 just deflects 5º ventrally from CdV1 due to the sigmoidal curvature of the tail.

IV) The wedged sacrum makes DV13 deflect 20º dorsally from CdV1 (Fig S4 B).

Girdles and appendicular skeleton

There has been some controversy on whether the pectoral girdle of sauropods would articulate in an "avian" fashion[42,43], that is, with the longest axis of the scapula sub-horizontal; or in a "crocodilian" fashion, that is, with the longest axis of the scapula sub-vertical. All evidence which seemed to point to a sub-horizontal scapula has been recently questioned[44–46], and a sub-vertical placement of the scapula (between 60º-70º) is favored based upon osteological[45,46] and myological evidence[44]. The pectoral girdle in sauropods has been recently reconsctructed with a sub-vertical scapula, the coracoid antero-ventral to the ribcage and most of the scapular head more anterior to the ribcage, since is the only possible position which allows: i) to keep the scapulocoracoids

articulated with the clavicles and interclavicle[46] and fit within the ribcage; ii) to not have the ribcage become an osteological stop for humerus retraction; iii) to have functional cingulo-axial and humeral musculature lines[44,47], particularly for *M. serratus* (*superficialis* and *profundus*), *M. sternocoracoideus, M. scapulohumeralis, M. subcoracoscapularis* and *M. deltoideus scapularis;* iv) to place the costo-coracoideal articulation subparallel to the distal ribs axis as is the case of all extant non-mammalian tetrapods[45]; v) to leave room dorsal to the distal expansion for the cartilaginous suprascapula, which would be the insertion point for *M. rhomboideus*[44,47].

The completeness of the pectoral girdle and anterior dorsal ribs of *Spinophorosaurus nigerensis* allowed testing different configurations with the inclinations of the scapulae and the connection of them with the other bones from the pectoral girdle (clavicles, interclavicle and coracoids) and with the dorsal ribs. Hypotheses for pectoral girdle-axial skeleton configurations were rejected if: i) the pectoral girdle would not fit the ribcage, ii) the pectoral girdle elements had to be disarticulated, iii) the lines of actions for cingulo-axial and humeral muscles were non-functional, iv) if the humerus could not be correctly articulated in the glenoid.

A subhorizontal placement of the scapula impedes articulating the clavicles and interclavicle with the acromia and coracoids respectively, as a subhorizontal placement separates the scapulocoracoids. It also creates inefficient lines of action for some muscles, such as the *M. deltoideus scapularis*[44]. The subvertical scapula of the 2009 reconstruction (Fig.4A), which has the acromia at the same height as the ventral edge of the dorsal vertebrae centra, also impedes articulating the pectoral girdle. Also, since the

length of the anteriormost 2 dorsal ribs reconstructed in 2009 was 25% shorter than the actual first two dorsal ribs of the *Spinophorosaurus* holotype, the glenoid would be situated more dorsal than the distal dorsal ribs. This impedes to articulate the humerus in a columnar, graviportal way and also creates an osteological stop for retracting the humerus. Also, placing the scapular head in articulation with the dorsal ribs creates an obstruction for *M. subcoracoscapularis pars scapularis*, which has its origin on the acromial region, on the medial side[44,47].

Only subvertical scapulae with the coracoid and the scapular head antero-ventral to the ribcage allow:

i. to correctly articulate the clavicles and interclavicles to the condition present in non-mammalian tetrapods (clavicles to acromia, both with interclavicle, interclavicle with coracoids and sternal plates[46]);
ii. to place the sternal plates parallel to the distal tips of the anterior dorsal ribs, so that they both can articulate with the sternal ribs as in extant archosaurs and lepidosaurs[45];
iii. to have room for a cartilaginous suprascapula with an homologous muscular and ligament attachment to the neural spines as in extant archosaurs[44,45];
iv. to articulate the forelimb so that the front feet step at the same medio-lateral distance from the sagittal plane of the skeleton than the hind feet, over the midline. Just as the ichnological evidence shows is the case narrow-gauge sauropod track-makers[48], since the pelvis of *Spinophorosaurus* is narrow and the feet of the reconstructed skeleton step over the midline.

All in all, it is the only position which the osteological, myological and ichnological evidence would not refute.

As for the pelvic girdle, the sacrum and preserved ilium suffered from compression as the rest of the axial skeleton, but the pubes and ischia were not heavily distorted. By articulating both pubes and ischia, the distance of the left and right articulation with the ilia helped in reconstructing a model close to the original width of the pelvis. The pelvis is narrow, and therefore the hind limbs articulate so that the hind feet can step over the midline of the dinosaur, as would be expected for an early branching non-neosauropod eusauropod[48].

As for the appendicular skeleton, it was articulated in ONP, then posed in a fast walking gait for the figures. The length of the speculative ulna and longest metacarpal of the virtual model are 730 mm and 283 mm, respectively, both values within the inferior ranges of estimated length for those bones (Table S2).

Physical model articulation

Taking advantage of rapid prototyping, a scaled 1:4 and 1:8 scale model of the neck of *Spinophorosaurus nigerensis* was used to test the results from the virtual reconstruction and range of motion analyses. The models were printed in an Ultimaker 2 printer, using thermoplastic Polylactic Acid (PLA), which can recreate surfaces with great accuracy. Physical scaled models have advantages and disadvantages next to the virtual models, but the most important advantage, however, is that the 3D printed model has the physical properties a virtual 3D model lacks, and hence osteological stops can be grasped in a way impossible in the virtual environment.

The 1:8 scale model was glued together with hot melted adhesive using the ONP criterion by fully articulating the pre- and postzygapophyses. The resulting model is very similar to the OIC achieved in the virtual environment, with only minor differences, not affecting the overall pose. The 1:4 model was employed to test the range of motion results (see below).

A 1:12 scale model of the hindlimb was also prototyped in order to assess the articulation of the tibia and fibula, and the articulation of both with the astragalus and femur.

6. Cervical range of motion analysis in *Spinophorosaurus nigerensis*

Cervical Ribs

Cervical ribs of sauropod dinosaurs have been considered a potential source of osteological stops or range of motion restriction[49]. Although most of them could not be reconstructed for this analysis, as they are still being prepared, recent analyses on ossified tendons (which is what the overlapping regions of the sauropod cervical ribs are according to histological analyses[50]) have shown their mechanical properties affected ranges of motion less than previously thought[51]. In fact, the histological analysis of several cervical ribs from distantly related sauropods (*Mamenchisaurus sp., Diplodocus sp.*) revealed that the collagen fibers were longitudinal[50]. This is evidence of a predominant tensile force acting on the ribs rather than compressive forces, and therefore higher dorsoventral mobility and more vertical neck positions[50]. It must be acknowledged that elongated cervical ribs may have somehow limited the range of motion of sauropod necks. Nevertheless, most non-diplodocid sauropods had the same type of elongated, overlapping cervical ribs, and they appear to have had the same type of histogenesis[50].

Therefore, since all sauropods with elongated cervical ribs would have had the same amount of motion limited by them, factors more variable, such as prezygapophyseal facet morphology, would have more effect on the amount of intervertebral motion in sauropods with elongated cervical ribs.

Measuring the range of motion

While there are standards for comparing neutral postures (See CNP, ONP, OIC, NBOP, etc.; above in "Terminology"), there are no standards defined yet for range of motion analyses: maximum articular excursions follow different criteria for different authors. Also, since some authors have employed either the real fossils, 1:1 scale casted replicas, virtual fossils or scaled down 3D printed replicas the results may slightly vary. Until more standardized criteria are established, range of motion analyses will necessarily need to be compared in relative terms, rather than absolute.

There has been a little controversy on how much pre- and postzygapophyseal articular facets can deflect before they effectively disarticulate. Early studies found the zygapophyseal safety factor (minimal overlap of the facets before there is too much strain on the zygapophyseal articular capsules *sensu* Stevens & Parrish[15]) to be at around 50% of overlap[16]. Nevertheless, observations on extant organisms such as crocodiles[14] or birds[22] have shown that alive extant animals can attain postures with barely any overlap between their zygapophyseal facets. Moreover, extant birds have zygapophyseal facets enlarged by a soft tissue capsule, which can increase the osteological facet up to 12.5%[25]. Knowing this, it could be argued that *in vivo* zygapophyseal facets were larger than their osteological counterparts, as the soft tissues did not fossilize. However, there is not a reliable way to estimate how much soft tissue is missing from the zygapophyses yet, and that soft tissue might have been variable between species and even among individuals from the same species.

All in all, it was decided to follow the protocol of Mallison[52], in which **vertebrae were deflected until only a minimum overlap of the facets was retained,** that is, just before they disarticulated (Fig. S5). That way, accounting for a larger facet *in vivo*, the range of motion is underestimated rather than overestimated (in accordance with what happens in present day archosaurs). The center of rotation was the anteriormost part of the cotyle of the posterior vertebra when the articulation was opisthocoelus and at midheight of the posterior centrum face in platycoelus articulations. This would prevent the misalignment of the neural canals, in order to avoid adopting poses that would put stress on the spinal cord (Heinrich Mallison pers. comm. 2016).

Criteria like the maximum dorso-ventral flexion employed by Christian[2] are less conservative and precise when it comes to comparing the ranges of motion of different taxa. According to Christian[2], it is in accordance with the results on the maximal dorsal excursion obtained for extant vertebrates with long necks. However, since the osteological stop may not happen in all taxa[15], unlike zygapophyseal disarticulation, the criterion is therefore less useful when it comes to comparative analyses.

7. Morphofunctional convergence at the cervicodorsal boundary between *Spinophorosaurus* and *Giraffa*

Cervicalization is a phenomenon widely recognized among sauropod dinosaurs, accounting as one of the main factors for neck elongation in several sauropod taxa[7,53] (such as many diplodocids, brachiosaurids and titanosaurs), although it is not the only mechanism of neck enlongation. The morphologic, ontogenetic and evolutionary processes of cervicalization are not completely understood. The study on anatomy and ontogeny of extant giraffes (*Giraffa camelopardalis*) and its comparison with its extant relative *Okapia johnstoni* and its extinct relatives shed some light on how the cervicalization process might occur in stages rather than in sudden transitions.

The first dorsal thoracic vertebra of giraffes has been claimed to be an eighth cervical vertebra of neoformation[15]. However, since the number of presacral vertebrae of giraffes is the same as that of okapis[54] and the brachial plexux position is identical in both giraffes and okapis, the neoformation hypothesis has been rejected, making the first thoracic vertebra of giraffes a partially cervicalized dorsal vertebra.

It is partially cervicalized because while it has some traits and functions of a cervical vertebra (See table S3 and Gungi & Endo[5]) it still retains one of the main characters and function of a thoracic vertebra, bearing the first functional rib of the ribcage, therefore supporting the viscerae.

In *Spinophorosaurus nigerensis* the cervicodorsal boundary happens to be functionally analogous to that of giraffes, with almost all the features of a cervical also present in its first dorsal which still has the thoracic rib, well differentiated from the

preceding cervical vertebra rib (Fig. 1). The analogies between giraffes and *S. nigerensis* are compared in Table S3 and Fig S6. These analogies converge on a greater dorso-ventral range of motion on the Last Cervical-First Dorsal and the First Dorsal-Second Dorsal joints of both animals than in their relatives without cervicalized vertebrae (Table 2). Therefore, the first dorsal vertebra of *S. nigerensis* is partially cervicalized and shows how the stages for the full cervicalization seen in other sauropods might have occurred, modifying the vertebra first while maintaining a plesiomorphic condition for the rib.

All in all, the cervicalization of dorsal vertebrae are part of the elongation of the neck process described in the evolutionary cascade of sauropod evolution[55,56] and related with the increased dorso-ventral range of motion of the neck, just as in giraffes[5].

8. Cervical range of motion in other sauropods

In order to compare how much the prezygapophyseal facet morphology might have affected inter-vertebral range of motion, other sauropod cervical vertebrae were digitized, articulated and their range of motion analyzed with the same protocol as for *Spinophorosaurus nigerensis*. The analysed specimens were *Camarasaurus sp.* (CM 584), *Camarasaurus grandis* (YPM 1905) and *Haplocanthosaurus priscus* (CM 572 and CM 879). Measurements of prezygapophyses and pictures were taken also for other eusauropods. Since other factors such as the distance from center of rotation to zygapophyseal facets[5] might affect the range of motion, only homologous positions at the cervicodorsal transition (the only which could be identified with certainty) were compared (Fig S5).

The prezygapophyseal facets of *Spinophorosaurus nigerensis* are more elongated than those *Haplocanthosaurus* or *Camarasaurus*, particularly in their laterals (Fig. S5), allowing a larger dorso-ventral osteological range of motion at the cervicodorsal boundary (27º between maximum dorsiflexion and ventriflexion) than the shorter prezygapophyses of *Camarasaurus sp.* (17º between maximum dorsiflexion and ventriflexion) and the rounded prezygapophyses of *Haplocanthosaurus* (20º between maximum dorsiflexion and ventriflexion in CM 572).

Illustrations of the zygapophyseal facets of both *Apatosaurus louisae* (CM 3018) and *Diplodocus carnegii* (CM 84) were employed by Kent Stevens in the software Dinomorph[17], where vertebrae were simplified to the zygapophyseal facets and distance

between those and the joint center of rotation[16,17]. The zygapophyseal facets of *A. louisae* were more elongated near the edges (similar to *Spinophorosaurus nigerensis*, albeit the facets are flat in *A. louisae* while the facets are convex and more elongated in *S. nigerensis*) than those of *D. carnegii*. Their analysis revealed *A. louisae* had more intervertebral osteological range of motion on its cervical vertebrae than *D. carnegii* (Figs 1 and 2 in Stevens and Parrish[16]). An independent analysis on the range of motion of *D. carnegii* revealed also less range of motion per joint in the dorso-ventral plane than *S. nigerensis* on the posterior half of the neck[19].

An analysis of the cervical vertebrae of Barosaurus (YPM 429, AMNH 6341) pointed its zygapophyseal facets were even shorter and wider than those of *D. carnegii,* suggesting a smaller dorso-ventral range of motion than in *D. carnegii* and more lateral flexibility[57].

The outline of the zygapophyseal facets therefore is correlated with intervertebral flexibility in the dorso-ventral and lateral planes. The third dimension of the zygapophyseal shape (whether zygapophyses are flat or convex) appears more related with bracing against torsional motion[17] than dorso-ventral and lateral motion.

Nevertheless, although more analyses need to be done in order to fully understand all factors contributing to intervertebral ranges of motion (including virtual reconstruction of muscles and ligaments), the outline of zygapophyseal facets is an important variable contributing to it: the longer the facet is anteroposteriorly, the more dorso-ventral flexibility.

9. Wedged sacra and forelimb length in sauropods

The reconstruction of the virtual *Spinophorosaurus nigerensis* skeleton shows the wedged sacrum has a key role in the geometry of the axial skeleton, contributing to the antero-dorsal sloping of the presacral vertebrae (Fig. 1, Fig. S4). The wedged sacrum, coupled with an elongation of the forelimb, makes the body plan of this sauropod very different from previous reconstructions (Fig. 4) and therefore high-browsing capabilities are more evident than before for basal eusauropods. The absence of wedged sacra on non-sauropod sauropodomorphs and non-eusauropod sauropods and the presence of wedged sacra on all examined eusauropods reveal it as a synapomorphy for at least Eusauropoda with important functional implications.

Many skeletal mounts and reconstructions of sauropods do not depict the strong impact the sacrum has on axial skeleton geometry and its correlation with forelimb elongation. Also, many sacra have been poorly figured (i.e. only in one view) or its fusion to the ilia has prevented studying their impact on the geometry of the axial skeleton. Nevertheless, some sauropod sacra had previously called attention upon their wedging/keystoning (most noticeably, that of *Mamenchisaurus youngii*[58]). The following taxa have been reconstructed and/or mounted previously, but little has been studied on how the wedged sacra should affect their skeletal reconstructions. Since "small inaccuracies can lead to drastic misinterpretations"[17] we review some specimens and their traditional interpretations and how the wedged sacra might affect their skeletal reconstructions.

Diplodocus

Several skeletal mounts of the Sheep Creek Quarry *Diplodocus carnegii* specimens excavated by the Carnegie Museum are on display on museums around the globe. Most of them have always been mounted with horizontal axial skeletons, even remounts based on a more recent understanding of sauropod body plans. The sacrum of both Carnegie Museum specimens (CM 84 and CM 94) is wedged in lateral view, up to 16º degrees of wedging in CM 94. Forelimbs are unknown in both Sheep Creek specimens. However, the wedging of their sacra would match the proportions of the humerus and femur found in other specimens of *Diplodocus*, such as USNM 10865[59].

A close examination of the mounted skeletons at Museum für Naturkunde (Berlin) and British Museum of Natural History (London), which were mounted according to our modern understanding of sauropod anatomy, still reveals some caveats, which clearly made their presacral vertebral series lower than their actual OIC. The dorsal series on both specimens were mounted disarticulated, on dorsals 2, 3 and 9 in the Berlin mount and on dorsals 3 and 8 in the London mount. The *Diplodocus* cast of the Naturmuseum Senckenberg (Frankfurt) was mounted with its axial skeleton almost completely articulated in osteologically neutral pose (with the exception of the last and penultimate cervical vertebrae, which are ventrally deflected), with the presacral vertebrae sloping dorsally. When mounting USNM 10865, Gilmore noticed articulating the presacral vertebrae with the sacrum made the presacral series slope antero-dorsally, with a change in deflection at the middle of the dorsal series, which created an arched back[59], although he did not specify the reason behind this (i.e. an obtuse wedged dorsal vertebra). The

arching of the back, however, is not present in the majority of *Diplodocus* mounts (which are reconstructed with a straight back in those specimens mounted in ONP).

This reveals that the body plan of *D. carnegii* and the morphofunctional "vacuum cleaning" models for the taxon should be revised. A virtual skeleton of *D. carnegii* for experimenting would greatly improve our understanding of the feeding capabilities of this taxon and would allow to further test ecological niches overlap hypotheses of the Morrison Formation sauropods.

Brachiosauridae

For a long time, Brachiosauridae sauropods were the only ones to be depicted with a dorsally sloping presacral column and interpreted as non-controversial high browsers, although there has been controversy on how high they might have browsed (Stevens[15] *contra* Christian & Dzemski[18]). Recent studies have hinted that the amount of sloping of the presacral series is crucial to delimit the height of the feeding envelopes of these sauropods (Fig. 4 in Stevens[15]). Although the focus of these studies focused on pectoral girdle orientation, sacral wedging is crucial to achieve a dorsal sloping of the presacral vertebrae series without deflecting the dorsal vertebrae. In order to test whether the highest angle proposed in Paul[13] was feasible, the sacrum and preserved axial column of the *Brachiosaurus altithorax* holotype (FMNH P25107) was digitized. The sacral wedging measured 29º (Fig. 2B), and is therefore compatible with the highest angle for the presacral vertebrae proposed in Stevens[15]. The illustration of the sacrum of *Giraffatitan brancai* in lateral view also reveals a quite high degree of wedging (around

26º-25º). As shown in Fig. 5 of Taylor, Wedel & Naish[20], a higher torso position for *Giraffatitan* allows a much taller maximum vertical reach. This wedging on the sacra of both taxa is compatible with studies of inter-vertebral discs stress, which predict a quite vertical, 60º-70º angle of the neck in ONP[18]: the cervicodorsal transition would have to be less dorsiflexed to achieve such postures than with a more horizontal dorsal series.

This implies that a greater sloping of the presacral vertebrae next to the extremely elongated forelimbs and necks in these taxa would provide these sauropods a greater verticalization of their feeding envelopes than in *Spinophorosaurus nigerensis*, and hence the maximum specialization of high browsing capabilities yet known in sauropod dinosaurs.

Mamenchisaurus youngi

That the sacrum of *Mamenchisaurus youngi* holotype (ZDM 0083), quite wedged particularly at the neural spines, was noticed soon after the publication of this taxon. Paul considered this sacrum as unique among sauropods[58]. The original skeletal reconstruction and mount had the axial skeleton disarticulated[58], and Paul identified the peculiar sacrum as crucial to the OIC of this sauropod. However, he considered that not only were the caudal and dorsal vertebrae deflected by the sacrum, but also that the pelvis was retroverted as in Camarasaurids[13]. Since pelvis retroversion and wedged sacrum are two different (though not mutually exclusive) conditions (Fig. S1) and other skeletal reconstructions of *Mamenchisaurus youngi* have the wedged sacrum but not the pelvic retroversion, further revision of the original fossils and their digitalization is advisable in

order to clarify whether it had or not pelvic retroversion. The wedged sacrum is compatible with the length of the forelimb of this taxon (Fig 2B).

Nevertheless, studies on *Mamenchisaurus sp.* reveal contradictory feeding ecological hypotheses. Paul[13] considers mamenchisaurs high browsers, whereas Christian et al[2] describe the neck of *Mamenchisaurus youngi* as quite stiff, with limited dorsiflexion, and proposes that it might have been a low browser feeding by laterally sweeping the neck (as in the "vaccum cleaner" hypothesis for diplodocids). Even though a more in depth morphofunctional analysis of this taxon is required, it too had a wedged sacrum, despite the different neck mobility next to other sauropods such as *Spinophorosaurus*.

Dicraeosaurus

Dicraeosauridae have some of the shorter necks among sauropod dinosaurs[53] and have traditionally been identified and depicted as low browsers or even ground-level browsers. *Dicraeosaurus hansemanni* preserves sacra and limb bones, albeit from different individuals. It is noticeable that it had short and robust humeri next to their femora. The sacrum is also wedged, albeit less than in most sauropods (Fig. 2B), but the dorsal vertebrae have obtuse wedging (Fig. 3, Fig. S2C), therefore counteracting the acute wedging of the sacrum within its overall osteologically induced curvature (as seen in Fig. 6.5C of Stevens & Parrish[14]). Although a detailed analysis should be carried out, it is evident that its osteologically induced curvature of the presacral vertebrae would make the head deflect a lot less dorsally than in other sauropods, radically different from the

OIC of *Spinophorosaurus nigerensis* and Brachiosauridae. The fact that the sacrum is wedged in an acute angle in an animal with an OIC that directs the skull toward the ground evidences the wedging of the sacrum is likely a plesiomorphy for dicraeosaurids.

Summing up, all known eusauropods with all kinds of neck lengths, cervical neural spine morphotypes (single or bifid), shorter or longer forelimbs, all kinds of tail lengths and all types of inferred feeding capabilities share a sacrum wedged more than 10º. The wedged sacrum has a pivotal role in determining the body plan (Fig. 1, Fig. S4), the vertical height of the feeding envelope (Fig. 2) and happens to correlate well with the forelimb length (the longer the humerus respect to the femur, the more wedged the sacrum, Fig. 3, Table 1). This makes the sacrum morphology directly related with the high browsing capabilities of many eusauropods. Despite this, the condition never reverts to the basal rectangular, non-wedged sacra of *Melanorosaurus readi* or *Kotasaurus yamanpalliensis* even in taxa with smaller forelimbs relative to their hindlimbs. This has three main implications: (i) the wedged sacrum becomes fixed within the sauropod phenotype (even in sauropods exploring feeding strategies different from high browsing, despite a sacrum which would make their presacral column inherently deflect dorsally), (ii) under a maximum parsimony interpretation of evolutionary processes, the condition is homologous and therefore plesiomorphic for all eusauropods, and (iii) the large functional impact the condition had on sauropod body plan and its fixation suggests an evolutionary innovation within the Eusauropod lineage.

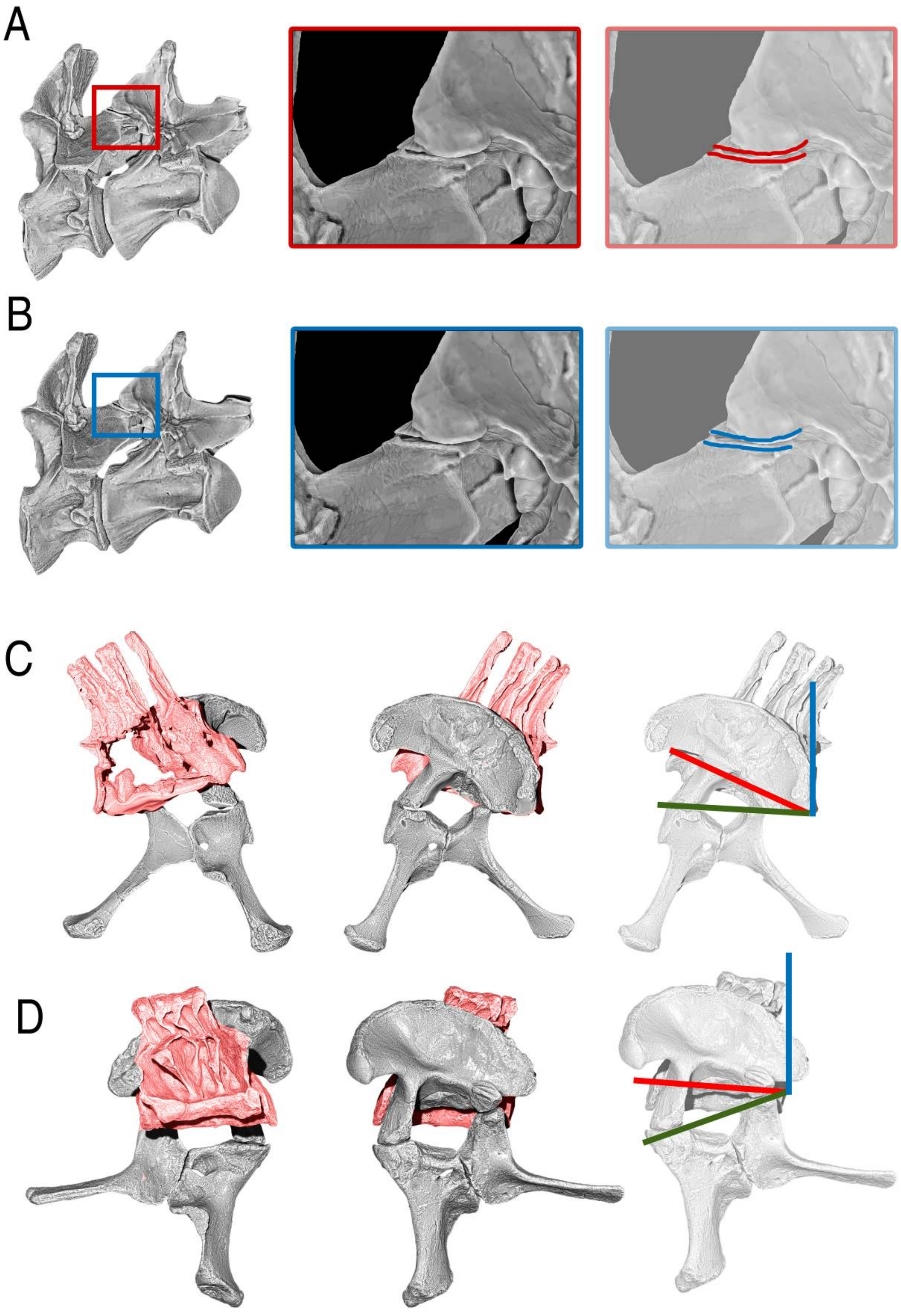
A
B
C
D

A – 3D model of first dorsal and last cervical vertebrae of *Spinophorosaurus nigerensis* in Osteological Neutral Pose (ONP, lateral view), showing the full zygapophyseal overlap in detail. Notice the central rims are not completely parallel.. B – First dorsal and last cervical vertebrae of *S. nigerensis* in Neutral Bone Only Posture (NBOP, lateral view). Notice how despite the central rims being parallel, the zygapophyses are not in 100% overlap as when they are in ONP. C – 3D model of sacrum and pelvis of *Diplodocus carnegii* (CM 94) right lateral and left lateral views (left and middle). D – 3D model of sacrum and pelvis of *Camarasaurus supremus* (AMNH 5761) in right lateral and left lateral views (left and middle). Notice how despite both having wedged sacra, the pelvis of *Diplodocus* is not retroverted, whereas the pelvis of *Camarasaurus* is. For C and D: blue = last sacral centra at 90º; red = sacricostal yoke; green: plane defined by ischial and pubic peduncles of the ilium. Fossils not to scale.

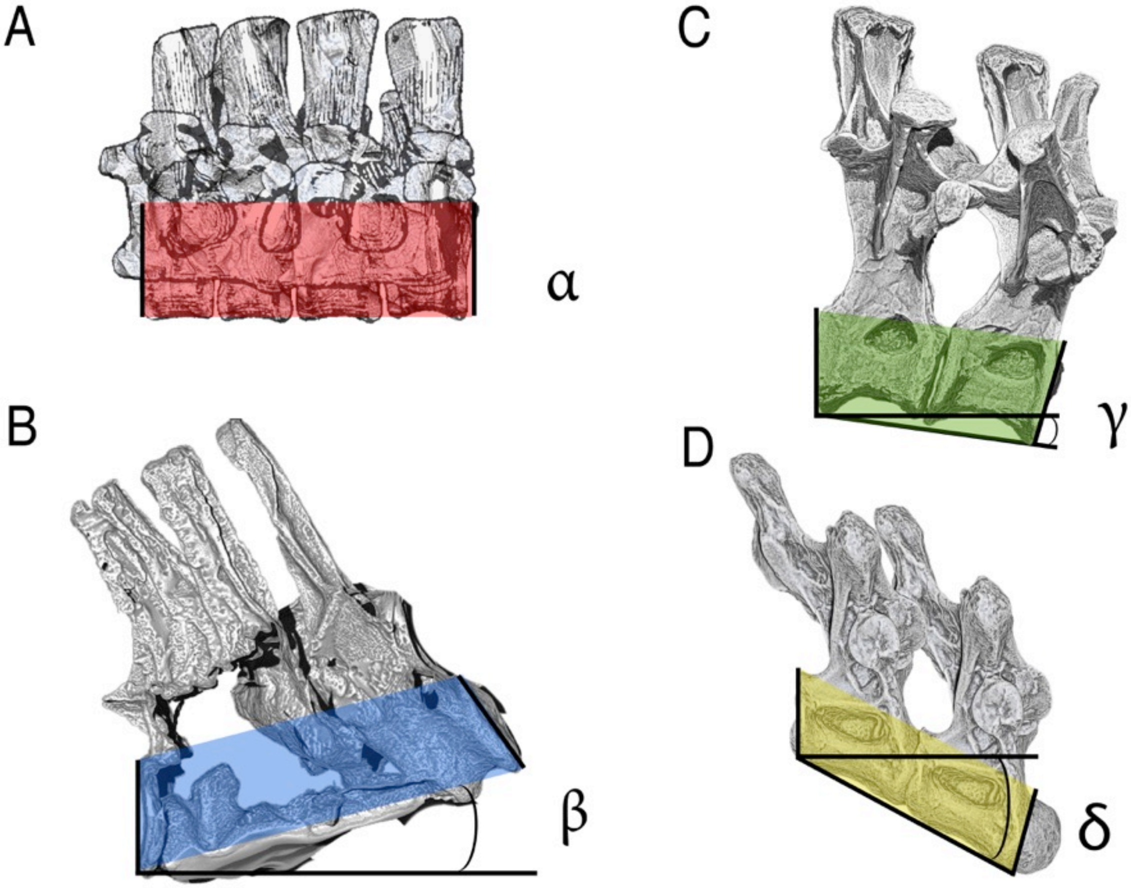

Fig. S2. Types of vertebral centra wedging in sauropod dinosaurs

A – Sacrum of *Kotasaurus* 26/S1Y/76 in left lateral view, showing no wedging at all B – 3D model of sacrum from *Diplodocus carnegii* (CM 94) in right lateral view, showing acute wedging (when the adjacent vertebrae form an acute angle). C – 3D model of two middle dorsal vertebrae of *Haplocanthosaurus priscus* (CM 572) in right lateral view, showing obtuse wedging. D – 3D model of two middle dorsal vertebrae of *Neuquensaurus australis* (MCS-5) in right lateral view, showing obtuse wedging due to the trapezoidal shape of the centra. Fossils not to scale.

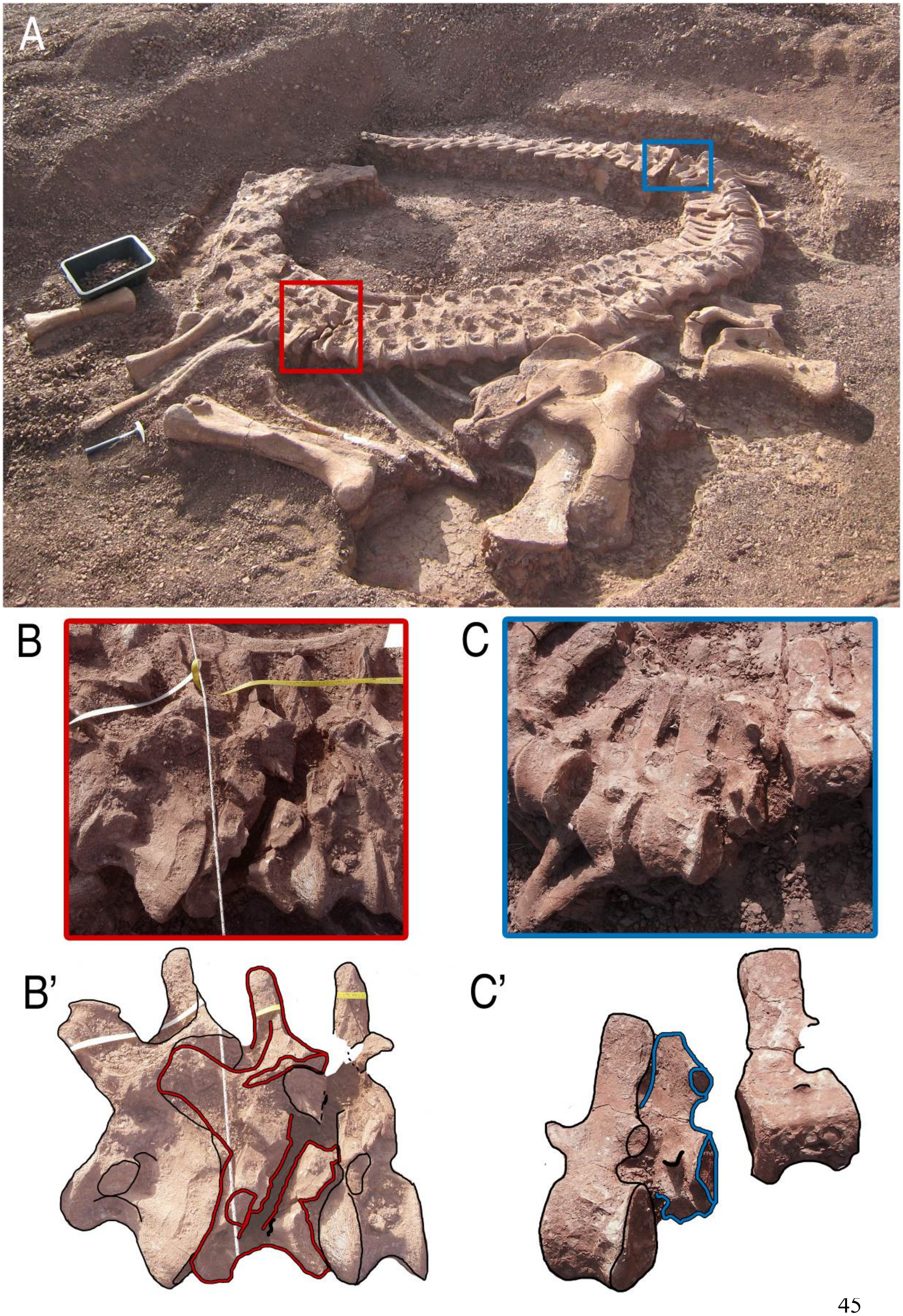
A
B
C
B'
C'

Fig. S3. *Spinophorosaurus nigerensis* holotype field quarry context.

A – General *in situ* situation of the holotype *Spinophorosaurus nigerensis* quarry during its excavation in Niger. B – Detail of the cervicodorsal transition *in situ*, damaged by a micro-fault, with the vertebrae outlined below in B'. C – Detail of caudal vertebra 15 *in situ*, damaged by the same micro-fault, with the vertebrae outlined below in C'.

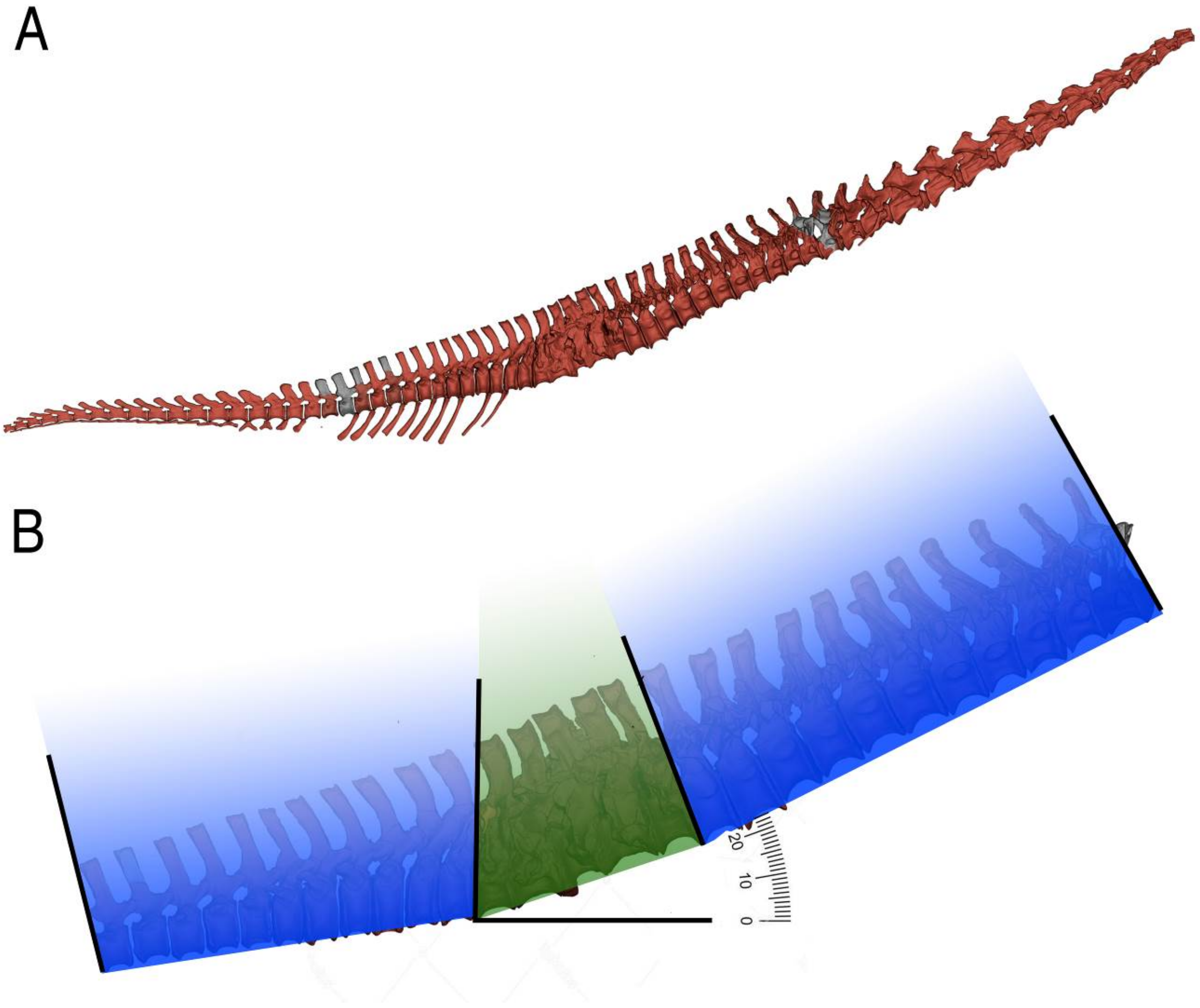

Fig. S4. Virtual *Spinophorosaurus nigerensis* vertebral column.

A – Osteologically induced curvature of the vertebral column of *Spinophorosaurus nigerensis* in lateral view. B – Detail on the sacrum of *S. nigerensis,* showing the 20º angle it makes deflect the dorsal vertebrae from the first caudal vertebra in lateral view.

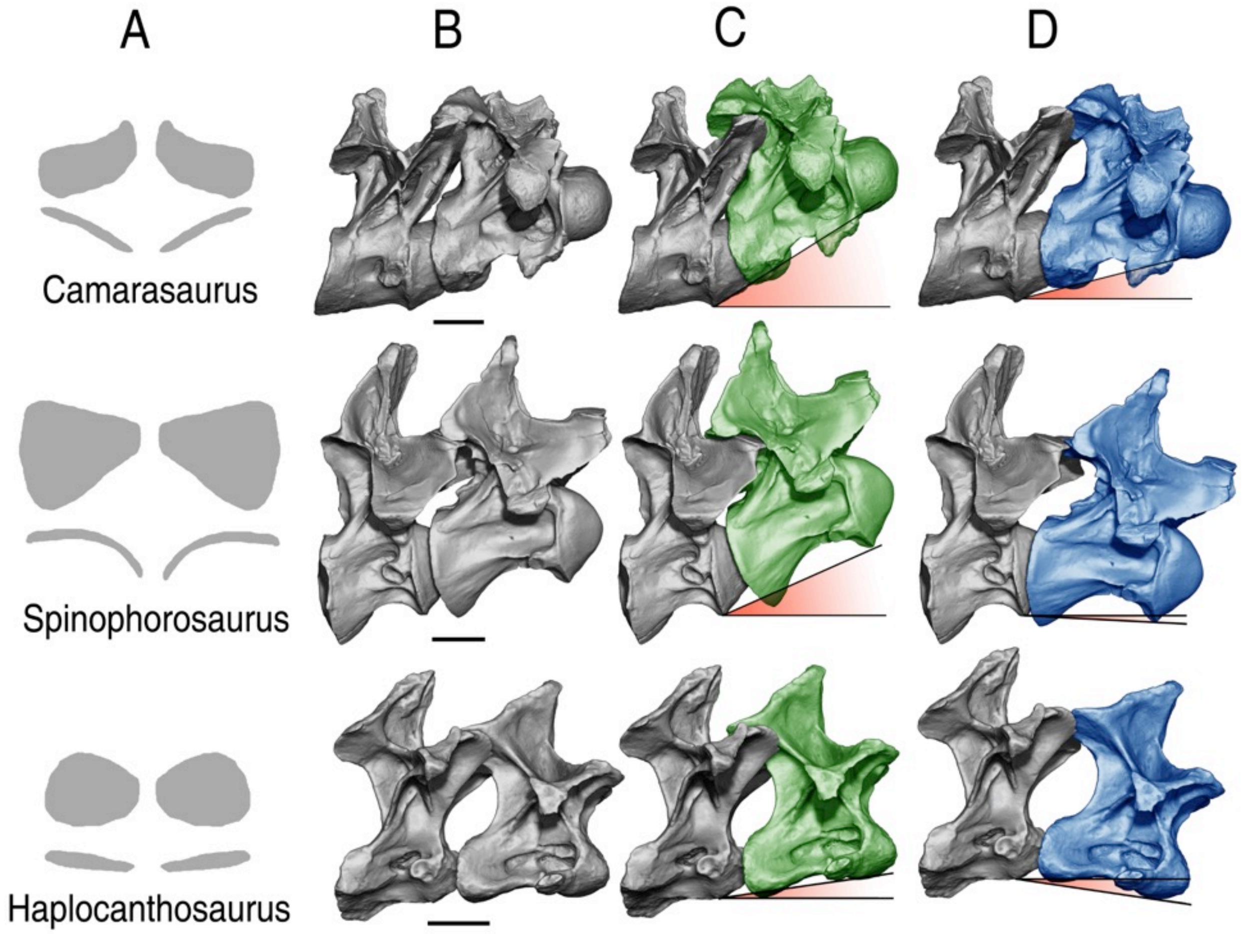

Fig. S5. Range of motion on the cervicodorsal transition in selected eusauropod sauropods

A – Outlines of the prezygapophyseal facets of *Camarasaurus sp.* (CM 584), *Spinophorosaurus nigerensis* (NMB-1698-R) and *Haplocanthosaurus priscus* (CM 572) in dorsal view. B – First dorsal and last cervical vertebrae of *Camarasaurus sp.* (CM 584), *Spinophorosaurus nigerensis* (NMB-1698-R) and *Haplocanthosaurus priscus* (CM 572) in Osteological Neutral Pose, right lateral view. C – Maximum dorsiflexion for that joint. D – Maximum ventriflecion at that joint. Scales under B = 100 mm.

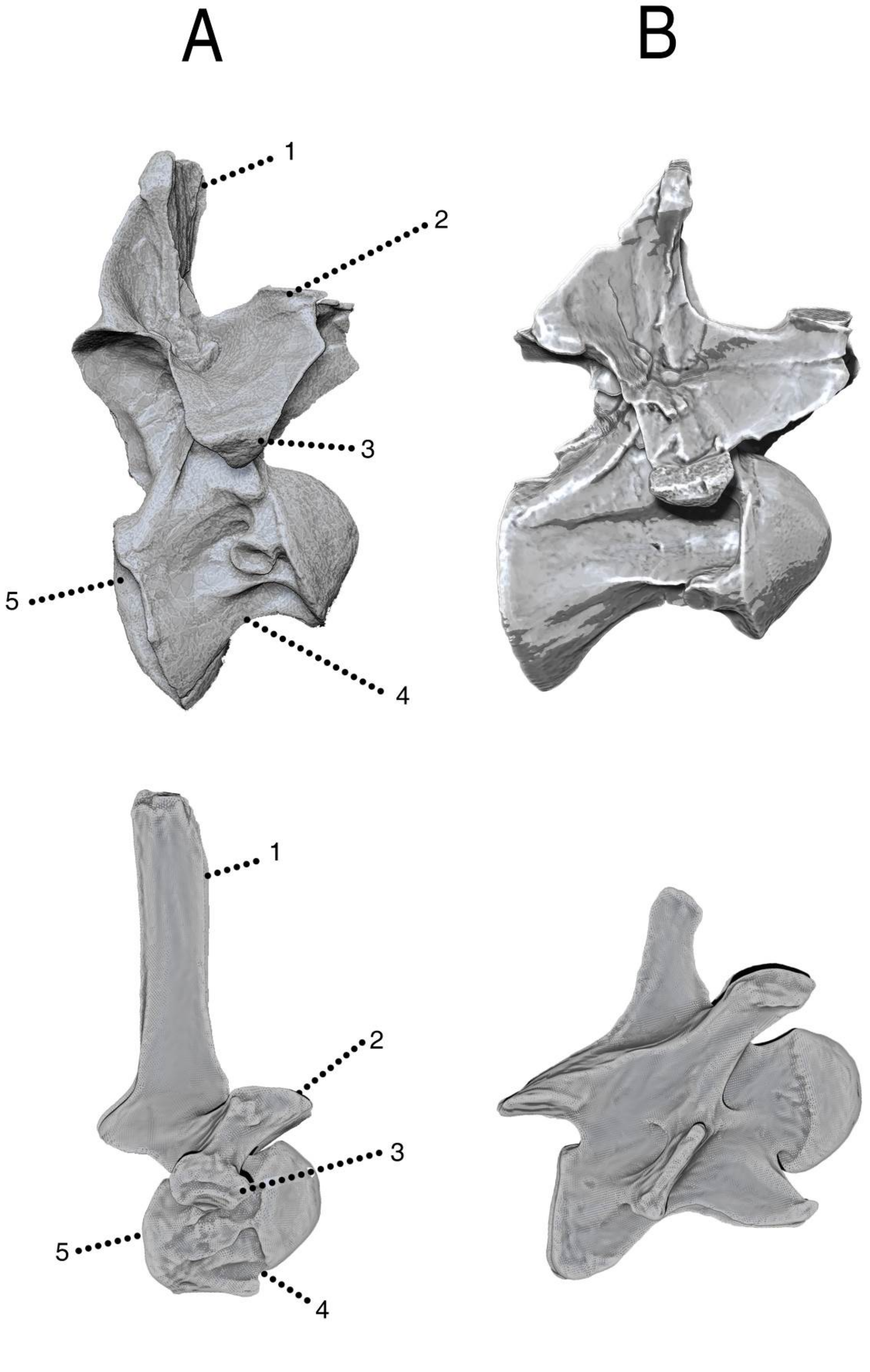
A
B
1
2
3
4
5
1
2
3
4
5

Fig. S6. Cervicalization in *Spinophorosaurus nigerensis* and *Giraffa camelopardalis*

A – First dorsal vertebra of *Spinophorosaurus nigerensis* (top) and *Giraffa camelopardalis* (bottom) in lateral view. B – Last cervical vertebra of *S. nigerensis.* (1) Neural spine with dorsal-like morphology; (2) prezygapophyseal rami and facets, cervical-like elongated; (3) dorsal rib ; (4) elongated centrum, in between the length of the remaining dorsal vertebra and preceding cervical vertebra; (5) asymmetric, trapezoid-like centrum, with the cotyle more dorsal than the condyle, cervical-like. Fossils and bones not to scale.

Table S1. Limb bone length ratios in several sauropod dinosaurs

One asterisk (*) indicates when ratios are estimated from bones stemming from different but similarly sized specimens. Two asterisks (**) indicates dubious measurements due to restoration not allowing to appreciate the actual length of the bone. Sc = Scapula. H = Humerus. U = Ulna. Mc = Longest Metacarpal.

**Species**	**H/Sc**	**U/H**	**Mc/U**	**F/H**
Spinophorosaurus nigeriensis NMB-1968-R	0.816	?	?	1.19*
Shunosaurus lii ZDM T5402	0.74	0.7	0.36	1.79
Patagosaurus fariasi MACN-CH 932 (juvenile)	0.83	0.74	?	?
Cetiosaurus oxoniensis OUMNH lectotype	0.91	0.73	?	1.28
Cetiosauriscus stewarti NHMUK R3078	?	0.80 **	?	1.44
Klamelisaurus gobiensis IVVP # V9492	0.70	0.65	?	1.30
Omeisaurus tianfuensis ZDM T5704	0.71-0.78	0.8	0.3	1.21-1.23
Omeisaurus jiaoi ZDM 5050	0.71	0.76	?	1.20
Mamenchisaurus youngii ZDM 0083	0.69	0.69	0.39	1,40
Ferganasaurus verzilini PIN-N-3042	?	0.71	0.35	1.21
Turiasaurus riodevensis CPT-1195 to CPT-1210	?	0.7	0.34	?
Mierasaurus bobyoungi UMNH VP.26004	?	?	0.44	?
Dicraeosaurus hansemanni MB.R.4886	?	0.66	?	1.96 *
Amargasaurus cazaui MACN-N-15	0.68	0.67	?	1.52
Apatosaurus louisae CM 3018	0.7	0.74	0.33	1.55
Diplodocus sp. USNM 10865	0.87	0.73	?	1,58
Giraffatitan brancai MB.R 2181 (formerly SII)	1.10	0.61	0.48	0.93*
Brachiosaurus altithorax FMNH P25107	?	?	?	0.99
Tehuelchesaurus benitezii MPEF-PV-1125	0.65	0.67	?	1.31
Camarasaurus grandis YPM 1901/1905 (subadults)	0.74	0.77	0,43	1,37
Camarasaurus lentus CM 11338 (juvenile)	0.73	0.74	0.42	1.36
Catethosaurus lewisii BYU 9047	?	0.76	0.41	?
Epachthosaurus sciuttoi UNPSJB-PV-920	?	0.69	0.52	
Dreadnoughtus schranni MPM PV 1156	0.91	0.63	?	1.19
Opisthocoelicaudia skarzynskii ZPAL MgD1/48	0.84	0.78-0.67	0.4	1.39

Table S2. Limb and pectoral girdle measurements and estimations in *Spinophorosaurus nigerensis* specimens

Estimated measurements are indicated with an asterisk (*) when based upon another specimen of *Spinophorosaurus nigerensis*, and two asterisks (**) when they are based upon other sauropod relatives. The ratios for the estimation of the missing elements were (i) Ulna = 0.7 (lowest) to 0.8 (highest) times Humerus length, (ii) Longest metacarpal = 0.35 (lowest) to 0.39 (highest) times Ulna length (see "Reconstruction of missing elements" above). Measurements in millimeters.

ELEMENT	LENGTH (PARATYPE)	LENGTH (HOLOTYPE)
Longest metacarpal	275-350**	248-316**
Ulna	785-897**	710-810**
Humerus	1121	1014*
Scapula	1374	1243

Table S3. Comparison of the cervicodorsal boundary between *Giraffa camelopardalis* and *Spinophorosaurus nigerensis*

Character states are as follows, for the following characters: 1) Short refers to centra that are taller than long. Long refers to centra that are longer than tall. Intermediate refers to square or close to square centra. 4) Wider refers to prezygapophyseal facets that are wider than long. Longer refers to prezygapophyseal facets that are longer than wide. 5) Large refers to the neural spine being as tall or taller than the vertebral centrum. Short refers to the neural spine being shorter than half the centrum height. Spino = *Spinophorosaurus nigerensis*; Giraffa = *Giraffa camelopardalis*

Character	DV 2	DV 1	Last Cervical	Penultimate cervical
1 - Length of vertebral centrum	Giraffa: **Short**<br>Spino: **Short**	Giraffa: **Intermediate**<br>Spino: **Intermediate**	Giraffa: **Long**<br>Spino: **Long**	Giraffa: **Long**<br>Spino: **Long**
2 - Posterior centrum morphology:	Giraffa: **Flat**<br>Spino: **Concave**	Giraffa: **Flat**<br>Spino: **Concave**	Giraffa: **Concave**<br>Spino: **Concave**	Giraffa: **Concave**<br>Spino: **Concave**
3 - Prezygapophyseal rami	Giraffa: **Reduced**<br>Spino: **Small**	Giraffa: **Small**<br>Spino: **Small**	Giraffa: **Long**<br>Spino: **Long**	Giraffa: **Long**<br>Spino: **Long**
4 - Prezygapophyseal facets	Giraffa: **Wider**<br>Spino: **Wider**	Giraffa: **Longer**<br>Spino: **Longer**	Giraffa: **Longer**<br>Spino: **Longer**	Giraffa: **Longer**<br>Spino: **Longer**
5 - Neural Spine Length	Giraffa: **Large**<br>Spino: **Intermediate**	Giraffa: **Intermediate**<br>Spino: **Intermediate**	Giraffa: **Short**<br>Spino: **Short**	Giraffa: **Short**<br>Spino: **Short**
6 - Ventral Keel	Giraffa: **Absent**<br>Spino: **?**	Giraffa: **Present**<br>Spino: **Present**	Giraffa: **Present**<br>Spino: **Present**	Giraffa: **Present**<br>Spino: **Present**

References for SI reference citations

1. Sereno, P. C. *et al.* Structural extremes in a cretaceous dinosaur. *PLoS One* **2**, 1–9 (2007).
2. Christian, A. *et al.* Biomechanical reconstructions and selective advantages of neck poses and feeding strategies of Sauropods with the example of *Mamenchisaurus youngi*. *PLoS One* **8**, 1–8 (2013).
3. Taylor, M. P. Quantifying the effect of intervertebral cartilage on neutral posture in the necks of sauropod dinosaurs. *PeerJ* (2014). doi:10.7717/peerj.712
4. Mateus, O., Maidment, S. C. R. & Christiansen, N. A. A new long-necked 'sauropod-mimic' stegosaur and the evolution of the plated dinosaurs. *Proc. R. Soc. B Biol. Sci.* **276**, 1815–1821 (2009).
5. Gunji, M. & Endo, H. Functional cervicothoracic boundary modified by anatomical shifts in the neck of giraffes. *R. Soc. Open Sci.* **3**, 150604 (2016).
6. Upchurch, P. The Evolutionary History of Sauropod Dinosaurs. *Philos. Trans. R. Soc. B Biol. Sci.* **349**, 365–390 (1995).
7. Wilson, J. & Sereno, P. C. Early Evolution and Higher Level Phylogeny of Sauropod Dinosaurs. *J. Vertebr. Paleontol.* **18**, 1–68 (1998).
8. Wedel, M. J., Cifelli, R. L. & Sanders, R. K. Osteology, paleobiology, and relationships of the sauropod dinosaur *Sauroposeidon*. *Acta Palaeontol. Pol.* **45**, 343–388 (2000).
9. Wedel, M. J., Cifelli, R. L. & Sanders, R. K. *Sauroposeidon proteles*, a new sauropod from the Early Cretaceous of Oklahoma. *J. Vertebr. Paleontol.* **20**, 109–114 (2000).

10. Chure, D., Britt, B. B., Whitlock, J. A. & Wilson, J. A. First complete sauropod dinosaur skull from the Cretaceous of the Americas and the evolution of sauropod dentition. *Naturwissenschaften* **97**, 379–391 (2010).

11. Erwin, D. H. Novelty and innovation in the history of life. *Curr. Biol.* **25**, 930–940 (2015).

12. Erwin, D. H. The topology of evolutionary novelty and innovation in macroevolution. *Philos. Trans. R. Soc. B Biol. Sci.* **372**, 1–8 (2017).

13. Paul, G. S. Restoring Maximum Vertical Browsing Reach in Sauropod Dinosaurs. *Anat. Rec.* **300**, 1802–1825 (2017).

14. Stevens, K. A. & Parrish, M. J. Neck posture, dentition, and feeding strategies in Jurassic sauropod dinosaurs. in *Thunder-lizards: The Sauropodomorph Dinosaurs* (eds. Tidwell, V. & Carpenter, K.) 212–232 (Indiana University Press, 2005).

15. Stevens, K. A. The articulation of sauropod necks: methodology and mythology. *PLoS One* **8**, 1–27 (2013).

16. Stevens, K. A. & Parrish, M. J. Neck Posture and Feeding Habits of Two Jurassic Sauropod Dinosaurs. *Science (80-.).* **284**, 798–800 (1999).

17. Stevens, K. A. DinoMorph: Parametric modeling of skeletal structures. *Senckenbergiana Lethaea* **82**, 23–34 (2002).

18. Christian, A. & Dzemski, G. Reconstruction of the cervical skeleton posture of *Brachiosaurus brancai* Janensch, 1914 by an analysis of the intervertebral stress along the neck and a comparison with the results of different approaches. *Foss. Rec. – Mitteilungen aus dem Museum für Naturkd.* **10**, 38–49 (2007).

19. Dzemski, G. & Christian, A. Flexibility Along the Neck of the Ostrich (*Struthio*

camelus) and Consequences for the Reconstruction of Dinosaurs With Extreme Neck Length. *J. Morphol.* **268**, 701–714 (2007).

20. Taylor, M. P., Wedel, M. J. & Naish, D. Head and Neck Posture in Sauropod Dinosaurs Inferred from Extant Animals. *Acta Palaeontol. Pol.* **54**, 213–220 (2009).

21. Mallison, H. The Digital *Plateosaurus* I : Body Mass , Mass Distribution and Posture Assessed Using Cad and Cae on a Digitally Mounted Complete Skeleton. *Palaeontol. Electron.* **2**, 1–26 (2010).

22. Mallison, H. The Digital *Plateosaurus* II: An Assessment of the Range of Motion of the Limbs and Vertebral Column and of Previous Reconstructions using a Digital Skeletal Mount. *Acta Palaeontol. Pol.* **55**, 433–458 (2010).

23. Carabajal, A. P., Carballido, J. L. & Currie, P. J. Braincase, neuroanatomy, and neck posture of *Amargasaurus cazaui* (Sauropoda, Dicraeosauridae) and its implications for understanding head posture in sauropods. *J. Vertebr. Paleontol.* **34**, 870–882 (2014).

24. Riesco, A., García-martínez, D. & Bastir, M. Reconstrucción del segmento torácico de la columna vertebral en paleoantropología. in *Life Finds a Way* (eds. Amayuelas, E. et al.) 241–244 (2018).

25. Taylor, M. P. & Wedel, M. J. The effect of intervertebral cartilage on neutral posture and range of motion in the necks of sauropod dinosaurs. *PLoS One* (2013). doi:10.1371/journal.pone.0078214

26. Reiss, S. & Mallison, H. Motion range of the manus of *Plateosaurus engelhardti* von Meyer , 1837. *Paleontol. Electron.* **17**, 1–19 (2014).

27. Paul, G. S. Terramegathermy and Cope's Rule in the Land of Titans. *Mod. Geol.* **23**, 179–217. (1998).

28. Jensen, J. A. A fourth new sauropod dinosaur from the Upper Jurassic of the Colorado Plateau and sauropod bipedalism. *Gt. Basin Nat.* **48**, 121–145 (1988).

29. McIntosh, J. S., Miller, W. E., Stadtman, K. L. & Gillette, D. D. The Osteology of *Camarasaurus lewisi* (Jensen, 1988). *BYU Geol. Stud.* **41**, 73–116 (1996).

30. Remes, K. *et al.* A new basal sauropod dinosaur from the middle Jurassic of Niger and the early evolution of sauropoda. *PLoS One* **4**, e6924 (2009).

31. Mallison, H. & Wings, O. Photogrammetry in paleontology - A practical guide. *J. Paleontol. Tech.* **12**, 1–31 (2014).

32. Vidal, D. & Díez Díaz, V. Reconstructing hypothetical sauropod tails by means of 3D digitization: *Lirainosaurus astibiae* as case study. *J. Iber. Geol.* 1–13 (2017). doi:10.1007/s41513-017-0022-6

33. Chatterjee, S. & Zheng, Z. Cranial anatomy of *Shunosaurus*, a basal sauropod dinosaur from the Middle Jurassic of China. *Zool. J. Linn. Soc.* **136**, 145–169 (2002).

34. Sereno, P. C. *et al.* Cretaceous sauropods from the sahara and the uneven rate of skeletal evolution among dinosaurs. *Science (80-.).* **265**, 267–271 (1999).

35. Royo-Torres, R. & Upchurch, P. The cranial anatomy of the sauropod *Turiasaurus riodevensis* and implications for its phylogenetic relationships. *J. Syst. Palaeontol.* **10**, 553–583 (2012).

36. Bonaparte, J. F. Les dinosaures (Carnosaures, Allosauridés, Sauropodes, Cétiosauridés) du Jurassique moyen de Cerro Condor (Chubut, Argentine). *Ann.*

Pal{é}ontologie **72**, 325–386 (1986).

37. Royo-Torres, R., Cobos, A. & Alcala, L. A Giant European Dinosaur and a New Sauropod Clade. *Science (80-.).* **314**, 1925–1927 (2006).
38. Zhang, Y. The Middle Jurassic dinosaur fauna from Dashanpu, Zigong, Sichuan Vol I. Sauropod dinosaurs (1). *Shunosaurus*. *Sichuan Publ. House Sci. Technol.* 1–89 (1988).
39. Woodward, A. S. On parts of the skeleton of *Cetiosaurus leedsi*, a sauropodous dinosaur from the Oxford clay of Peterborough. *Proc. Zool. Soc. London* 232–243 (1905).
40. Alifanov, V. & Averianov, A. O. *Ferganasaurus verzilini*, gen. et sp. nov., a new neosauropod (Dinosauria, Saurichia, Sauropoda) from the Middle Jurassic of Fergana Valley, Kirghizia. *J. Vertebr. Paleontol.* **23**, 358–372 (2003).
41. Carpenter, K., Madsen, J. & Lewis, A. Mounting of fossil vertebrate skeletons. in *Vertebrate Paleontological Techniques vol. 1* (eds. Leiggi, P. & May, P.) 285–322 (Cambridge University Press, 1994).
42. McIntosh, J. S. Sauropoda. in *The Dinosauria* (eds. Weishampel, D. B., Dodson, P. & Osmólska, H.) 345–401 (University of California Press, 1990).
43. Upchurch, P., Barrett, P. M. & Dodson, P. Sauropoda. in *The Dinosauria, 2nd edition* (eds. Weishampel, D. B., Dodson, P. & Osmólska, H.) 259–322 (University of California Press, 2004).
44. Remes, K. Evolution of the Pectoral girdle and Forelimb in Sauropodomorpha (Dinosauria, Saurischia): Osteology, Myology and Fuction. *Evolution* (Ludwig-Maximilians-Universität München, 2007). doi:10.1002/ajpa.1330170113

45. Schwarz, D., Frey, E. & Meyer, C. A. Novel reconstruction of the orientation of the pectoral girdle in sauropods. *Anat. Rec.* **290**, 32–47 (2007).

46. Tschopp, E. & Mateus, O. Clavicles, interclavicles, gastralia, and sternal ribs in sauropod dinosaurs: New reports from Diplodocidae and their morphological, functional and evolutionary implications. *J. Anat.* **222**, 321–340 (2013).

47. Hohn-Schulte, B. Form and Function of the Shoulder Girdle in Sauropod Dinosaurs: A Biomechanical Investigation with the Aid of Finite Elements. (Ruhr-Universität Bochum, 2010).

48. Carrano, M. T. The Evolution of Sauropod Locomotion: Morphological Diversity of a Secondarily Quadrupedal Radiation. in *The Sauropods: Evolution and Paleobiology* (eds. Curry Rogers, K. & Wilson, J. A.) 229–249 (University of California Press, 2005).

49. Martin, J., Martin-Rolland, V. & Frey, E. Not cranes or masts, but beams: the biomechanics of sauropod necks. *Oryctos* **1**, 113–120 (1998).

50. Klein, N., Christian, A. & Sander, P. M. Histology shows that elongated neck ribs in sauropod dinosaurs are ossified tendons. *Biol. Lett.* **8**, 1032–1035 (2012).

51. Persons, W. S. & Currie, P. J. Dragon Tails: Convergent Caudal Morphology in Winged Archosaurs. *Acta Geol. Sin.* **86**, 1402–1412 (2012).

52. Taylor, M. P. & Wedel, M. J. Why sauropods had long necks; and why giraffes have short necks. *PeerJ* **1**, 1–41 (2013).

53. Rauhut, O. W. M., Remes, K., Fechner, R., Cladera, G. & Puerta, P. Discovery of a short-necked sauropod dinosaur from the Late Jurassic period of Patagonia. *Nature* **435**, 670–672 (2005).

54. Solounias, N. The remarkable anatomy of the giraffe's neck. *J. Zool.* **247**, 257–268 (1999).

55. Sander, P. M. *et al.* Biology of the sauropod dinosaurs: The evolution of gigantism. *Biol. Rev.* **86**, 117–155 (2011).

56. Sander, P. M. An evolutionary cascade model for sauropod dinosaur gigantism--overview, update and tests. *PloS one* (2013). doi:10.1371/journal.pone.0078573

57. Taylor, M. P. & Wedel, M. J. The neck of *Barosaurus*: longer , wider and weirder than those of Diplodocus and other diplodocines. *PeerJ Prepr.* **4:e67v2**, (2016).

58. Paul, G. S. *Mamenchisaurus youngi*, the dorky dinosaur. *Prehist. Times* **83**, 20–21 (2007).

59. Gilmore, C. W. On a newly mounted skeleton of *Diplodocus* in the United States National Museum. *Proc. United States Natl. Museum* **81**, 1–21 (1932).
